# A Map of Copy Number Variations in Chinese Populations

**DOI:** 10.1371/journal.pone.0027341

**Published:** 2011-11-07

**Authors:** Haiyi Lou, Shilin Li, Yajun Yang, Longli Kang, Xin Zhang, Wenfei Jin, Bailin Wu, Li Jin, Shuhua Xu

**Affiliations:** 1 Chinese Academy of Sciences Key Laboratory of Computational Biology, Chinese Academy of Sciences and Max Planck Society (CAS-MPG) Partner Institute for Computational Biology, Shanghai Institutes for Biological Sciences, Chinese Academy of Sciences, Shanghai, China; 2 Ministry of Education (MOE) Key Laboratory of Contemporary Anthropology, School of Life Sciences and Institutes of Biomedical Sciences, Fudan University, Shanghai, China; 3 Children's Hospital Boston, Harvard Medical School, Boston, Massachusetts, United States of America; Emory University School Of Medicine, United States of America

## Abstract

It has been shown that the human genome contains extensive copy number variations (CNVs). Investigating the medical and evolutionary impacts of CNVs requires the knowledge of locations, sizes and frequency distribution of them within and between populations. However, CNV study of Chinese minorities, which harbor the majority of genetic diversity of Chinese populations, has been underrepresented considering the same efforts in other populations. Here we constructed, to our knowledge, a first CNV map in seven Chinese populations representing the major linguistic groups in China with 1,440 CNV regions identified using Affymetrix SNP 6.0 Array. Considerable differences in distributions of CNV regions between populations and substantial population structures were observed. We showed that ∼35% of CNV regions identified in minority ethnic groups are not shared by Han Chinese population, indicating that the contribution of the minorities to genetic architecture of Chinese population could not be ignored. We further identified highly differentiated CNV regions between populations. For example, a common deletion in Dong and Zhuang (44.4% and 50%), which overlaps two keratin-associated protein genes contributing to the structure of hair fibers, was not observed in Han Chinese. Interestingly, the most differentiated CNV deletion between HapMap CEU and YRI containing *CCL3L1* gene reported in previous studies was also the highest differentiated regions between Tibetan and other populations. Besides, by jointly analyzing CNVs and SNPs, we found a CNV region containing gene *CTDSPL* were in almost perfect linkage disequilibrium between flanking SNPs in Tibetan while not in other populations except HapMap CHD. Furthermore, we found the SNP taggability of CNVs in Chinese populations was much lower than that in European populations. Our results suggest the necessity of a full characterization of CNVs in Chinese populations, and the CNV map we constructed serves as a useful resource in further evolutionary and medical studies.

## Introduction

Copy number variation (CNV) is a type of global genetic variations in human genome, defined as a segment of DNA larger than one kilobase presenting copy-number differences by comparison of two or more genomes [1]–[4]. One single or co-effects of multiple genomic rearrangements such as deletion, insertion, duplication and unbalanced translocation are likely to cause CNVs. By changing gene dosage, interrupting coding sequences, and influencing neighboring gene regulation, CNVs can impact on gene expression and phenotypes [5]–[7]. It is also known that CNVs are associated with several complex neurological diseases including Autism and Schizophrenia [8]–[10], and the susceptibility to other complex traits such as HIV, Crohn's disease and psoriasis [11], [12].

As a source of human genetic diversity, with various genetic patterns of nucleotide and structural variability in human populations [13], [14], CNV is vital in understanding the distribution in different populations and it is indispensable to find out their roles in conveying disease risk. A few studies have used various approaches to explore CNVs in different populations during the past seven years [2], [4], [15]–[18]. Other than the discovery of Han Chinese samples in HapMap project [2], [16], [19], [20], there are a certain number of studies reporting CNVs discovery in Han Chinese population [6], [21]–[24]. However, none of these studies focused on the minority of Chinese populations. Given the fact that there are fifty-five minority ethnic groups in China, the results of Chinese CNV map could hardly be complete without considering their genetic diversities. In this study, we applied Affymetrix SNP 6.0 which was designed for both SNP and CNV detection to explore genome wide CNVs in both Han Chinese and other six Chinese minority ethnic groups. With over one hundred samples from seven ethnic groups, we generated a comprehensive Chinese CNV map and performed gene ontology analysis on CNV overlapping genes. We found that CNV diversity varies at population level. The genetic relationship among ethnic groups is recovered by constructing a phylogenetic tree based on our devised genetic distance. In addition, we performed CNV sharing analysis to find genomic distribution patterns of CNVs among populations. We also estimated population differentiation by calculating F_ST_ based on the inferred allele frequency. Moreover, we showed that population structure can be detected by biallelic CNPs,but only about twenty-five percent were well tagged by flanking SNPs. Finally, we conducted population-specific CNV analysis, and identified a number of CNV candidates showing significant differentiated signals among populations which might be under selections.

## Results

### CNV detection

In this study, a total of 155 samples performed by the Affymetrix Genome-Wide Human SNP Array 6.0 have passed a series of quality control in CNV detection, comprising 80 Han Chinese, 41 Tibetans, 9 Dongs, 8 Yaos, 6 Zhuangs, 8 Lis, and 3 Uyghurs. By using Birdsuite algorithm [25] to genotype CNPs and detect rare CNVs, we discovered 21,840 CNV events in total, (18,306 deletions and 3,534 duplications), more than 90% of which were overlapping with pre-defined CNP loci [16]. The average number of CNV events per individual was 140.9 (118.1 for deletions and 22.8 for duplications) after combining the results of Canary and Birdseye and removing the inconsistent calls between the two algorithms. Furthermore, the average deletion count per individual was near 5 times that of duplication count, however, the length of single duplication event (median 49,801 bp) was much larger than that of single deletion event (median 8,823 bp)(Table 1, Figure S2).

**Table 1:** Summary of CNV detection in Chinese population.

	Han	Tibetan	Dong	Yao	Li	Zhuang	Uyghur	overall
sample size	80	41	9	8	8	6	3	155
**deletion count per individual**								
average	119.2	122.3	109.4	104.9	108.9	106.5	139.7	118.1
s.d.	8.8	11.8	14.5	9.4	5.9	11.4	14.2	11.7
**duplication count per individual**								
average	22.9	23.1	24.1	21.5	20.3	19.8	24.3	22.8
s.d.	6.7	9	6.5	5.2	4.7	1.8	3.2	7.1
**total CNV count per individual**								
mean	142.1	145.3	133.6	126.4	129.1	126.3	164	140.9
s.d.	11.2	15.5	18.6	10.5	5	11.2	12.3	14.3
**single CNV event length (median bp)**								
deletion	8,894	8,459	9,086	9,122	8,894	8,894	7,792	6,395
duplication	48,615	54,811	49,801	55,742	49,801	43,046	46,796	26,146
total CNV	10,576	10,255	10,812	11,476	11,119	10,302	10,011	7,512
**total CNV event length per individual (median bp)**								
deletion	3,386,738	3,633,613	3,042,041	2,579,284	3,300,673	2,899,222	3,868,875	3,394,127
duplication	2,511,063	2,562,481	1,760,324	2,268,591	1,733,213	1,819,230	2,175,929	2,296,873
total CNV	5,942,010	6,191,108	5,063,012	4,720,480	5,440,737	4,917,234	6,709,799	5,812,415

CNVs observed in more than one individual with any amount of overlap were classified as non-singleton CNVs while those detected in only one individual were classified as singleton CNVs. Non-singleton CNVs dominated (96.7%) in the detection of all CNVs, and the total 732 singletons consisted of 390 deletions (2.1% of copy loss events) and 342 duplications (9.6% of copy gain events). The proportion of singleton duplications was much higher than that of singleton deletions (p<2.2e-16, Fisher exact test). Additionally, since there were five replicate samples in this study, we can compare the CNP and SNP calling concordance between each pair of replicates. The average consistency of calling for CNP and SNP were 99.71% and 99.91%, respectively, which indicated the good reproducibility of the experiment.

### CNV map of Chinese populations

The average number of individual CNV events varied among these groups (Table 1). Han and Tibetan had averagely 142 and 145 CNVs per individual, while in Li, Dong, Yao and Zhuang, these numbers were only around 130, but excessively high (164) for Uyghur probably due to its genetic admixture background [26]–[28]. All these groups were included in the Chinese CNV map building.

**Figure 1:**
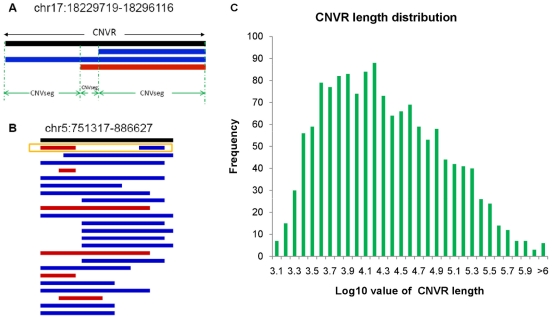
Copy Number Variable Region (CNVR) and Copy Number Variable Segment (CNVseg). Due to the inconsistent calling of CNV boundaries, CNVR refers to a union of overlapping CNVs while a CNVseg represents the minimal units of CNV. In the two panels, black bars denote CNVR, below which each bar denote a CNV call in one individual. Red and blue bars represent deletion and duplication, respectively. (A). An example of CNVR and CNVsegs on chromosome 17 starting from 18,229,719 to 18,296,116. Dashed lines (green) dissect CNVR into small contiguous CNVsegs. (B). An example of a complex CNV region. The rectangle (orange) points out that one individual has both deletion and duplication in the region, to which we refer as complex CNVR in this study. (C). CNVR length distribution. Length distribution of 1440 CNVRs constructed in this study ranges from 1.03 kb to 1.73 Mb.

By extending the boundaries of overlapping CNVs in different individuals, that is, the union region of any amount of overlapping CNVs which we called Copy Number Variable Region (CNVR) in the study (Figure 1A), we have identified 1440 CNVRs by combining the results of CNPs and rare CNVs from 155 Chinese samples (Table S1). In this CNVR map, 708 regions were comprised of non-singleton CNVs and 835 CNVRs overlapped with predefined CNP loci [16]. CNVRs ranged from 1.06 kb to 1.73 Mb (with a median and average size of 15.4 kb and 59.2 kb, respectively; Figure 1C) and spanned ∼3.0% of human genome. The genomic distribution of 1440 autosomal CNVRs was shown in Figure S3 and Table S1, and individual CNV events on the non-singleton CNVRs were shown in Figures S6 and S7.

**Figure 2:**
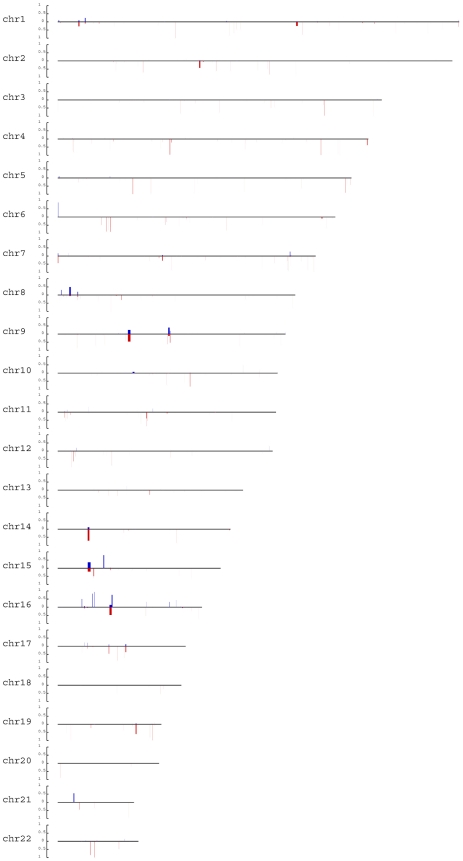
Genomic CNV frequency distribution. The height of bars indicates CNV frequency. Red and blue bars represent deletion and duplication respectively.

We assigned each CNVR a genotype with a copy state occupying the largest length in that region (Methods). Figure 2 shows the frequency of genomic CNV events according to the assigned genotypes. Among them, 257 regions had frequency larger than 10%. Due to the complexity of multiple CNVs of the same individual in one CNVR (Figure 1B), we dissected CNVR into small segments by cutting the two breakpoints of each CNV on the CNVR, which we called ‘CNVseg’ hereafter (Figure 1A). The genotype of CNVsegs can be easily assigned as the copy state of the segment on that region without introducing any inconsistency. There were totally 2861 CNVsegs in our study, which would be useful in further analysis of fine-scale mapping (Table S1).

We also compared CNVRs in this study with previously reported CNVs in the Database of Genomic Variants (DGV, August 2009 v8) (http://projects.tcag.ca/variation). A total of 235 CNVRs were not overlapped with any amount of CNV loci in DGV, including 224 non-singleton CNVs and 11 singleton CNVRs. However, these numbers might be underestimated, as many previous studies used low-resolution experiment platform providing rougher boundaries of CNVs [16], [29]. Furthermore, we made a comparison with HapMap Phase 3 data which were performed as the same workflow as our data (Methods). There were 433 CNVRs in our analysis undiscovered in HapMap Phase 3 data, including 332 singleton CNVs and 101 non-singleton CNVRs. To sum up, the remaining CNVRs previously unidentified in either DGV or HapMap were comprised of 170 singleton CNVs and 5 non-singleton CNVRs.

There were 1079 genes in RefSeq database located within 618 of the 1440 CNVRs (Table S1). Among these identified genes, 448 and 323 genes had only copy number gains (duplication group) and losses (deletion group), respectively, and the other 308 genes had both (multi-allelic group). According to variant type of gain, loss or multi-allelic, we performed gene ontology analysis to explore the enrichment in GO categories of these CNVR overlapping genes in the three groups by running the Gene Ontology Tree Machine [30] respectively. Although the enrichment contents varied among the three groups, there is a common pattern – a paucity of enrichment in cellular component compared with biological process and molecular function, and the bias was more significant in duplication and multi-allelic group. (Table S2) (p<0.01, Chi-square Test).

### Population analysis of CNVs

#### Population genetic diversity

**Figure 3:**
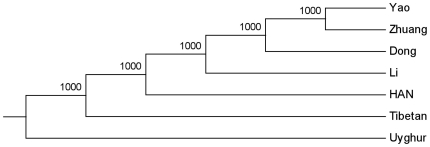
Phylogenetic tree of Chinese ethnic groups constructed by UPGMA. Phylogenetic tree of Chinese ethnic groups based on average pairwise genetic population distance between ethnic groups with 1,000 bootstrap replications by UPGMA.

CNV diversity can be reflected by the average number of individual CNV events among ethnic groups (Table 1). We asked whether such diversity was able to capture the genetic relationship among those groups. In order to delineate the genetic distance between two populations, we devised a measure based on the average number of different CNV genotype callings between two unrelated individuals from different populations (Methods). The pairwise comparisons were performed both among and within seven ethnic groups for all CNVRs. Yao and Uyghur had 122.1 and 173 of 1440 CNVRs–the smallest and biggest number of different loci from two individuals within populations on average, respectively (Table 2). The genetic distance between Yao and Zhuang was the smallest (126.8 loci on average) while Uyghur and Tibetan differed the most (176.2 loci on average). Based on the genetic distance, we constructed a phylogenetic tree (Figures 3, S4). Ethnic groups such as Yao, Zhuang, Dong and Li, with relative small geographic distance, were closer to each other than to other populations. The topology of the phylogenetic tree was not only under our expectation but also reflected a linguistic pattern among 7 Chinese ethnic groups, which suggests that ethnic groups sharing the same language family have relative small CNV genetic distance.

**Table 2:** Pairwise genetic distance among Chinese ethnic groups.

Ethnic groups	Dong	HAN	Li	Tibetan	Uyghur	Yao	Zhuang
**Dong**	142.8	--	--	--	--	--	--
**HAN**	151.9	150.2	--	--	--	--	--
**Li**	145.9	148.4	130.2	--	--	--	--
**Tibetan**	157.3	154.1	151.0	151.4	--	--	--
**Uyghur**	171.8	174.2	162.2	176.2	173.0	--	--
**Yao**	134.8	145.2	140.6	150.4	165.8	122.1	--
**Zhuang**	137.2	145.9	139.0	151.1	168.8	126.8	129.1

#### CNV sharing analysis

**Figure 4:**
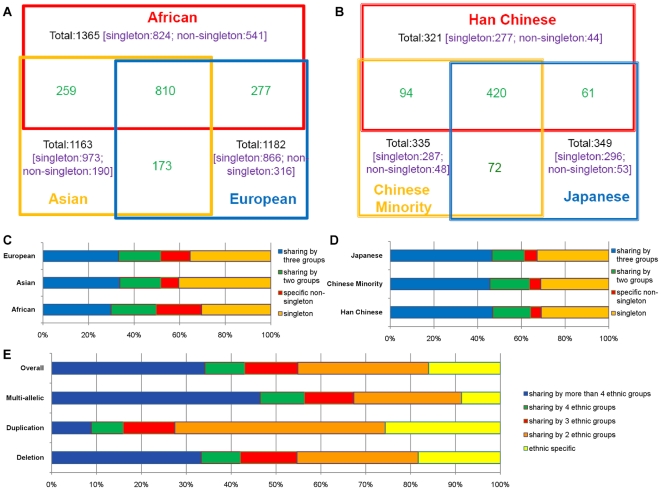
CNV sharing. Venn diagram (A) and bar chart (C) of CNV sharing results among African, Asian and European groups (each group with sample-size 425). Venn diagram (B) and bar chart (D) of CNV sharing results among Han Chinese, Chinese minority and Japanese groups (each group with sample-size 75). (E). Bar chart of sharing results in 708 non-singleton CNVRs (333 deletion CNVRs, 113 duplication CNVRs and 254 multi-allelic CNVRs) among 7 Chinese ethnic groups.

We conducted CNV sharing analysis to further investigate the distribution pattern of CNV regions among different populations. If one CNVR contains CNVs from more than one population, then this CNVR was labeled as ‘sharing’. After combining HapMap samples and adjusting the sample-size by random sampling among the populations, we calculated the average CNV sharing counts of three world-wide major population groups (African, European and Asian) and three within-Asian groups (Han Chinese, Chinese minority and Japanese), respectively. It showed that African has more specific CNVs than Asian or European, and the number of CNVRs shared by Asian and European was smaller than that shared either by Asian and African or by European and African (Figures 4A, S8A, S8C, S8E). On the other hand, the shared CNVRs within Chinese populations were more than those between any Chinese population and Japanese population, indicating that the genetic relationship within Chinese ethnic groups was closer to each other than to other populations (Figures 4B, S8B, S8D, S8F). Overall, the proportion of regions shared by at least two groups rises to 50% for world-wide groups, and up to 60% for within-Asian groups (Figure 4C and 4D). Such relationship was also observed in pairwise CNV sharing, where populations from different continents share ∼40% while populations within the same continent shared ∼50% except ASW (Table 3). Both results showed that multi-allelic CNVRs have much higher proportion in CNVRs sharing than in population specific CNVRs, which was more significant for the regions shared by all three groups in the Venn diagram (Figure S8). Furthermore, we focused the sharing pattern of 708 non-singleton CNVRs identified within our Chinese ethnic groups. Over 80% of the non-singleton CNVRs was shared by more than 2 ethnic groups, though compared with deletion and multi-allelic CNVRs among Chinese ethnic groups, non-singleton duplication CNVRs were shared by fewer samples and had relative larger proportion of singleton duplication (Figure 4E). In conclusion, CNV sharing commonly exists among ethnic groups to the extent of contributing more than half of CNV regions in each population and this sharing proportion increases as the genetic distance decreases.

**Table 3:** Pairwise CNVR sharing among different populations*.

Population	ASW	CEU	GIH	CHH	JPT	LWK	MEX	MKK	CHM	TSI	YRI
**ASW**	-	0.565	0.499	0.531	0.523	0.596	0.612	0.585	0.587	0.543	0.644
**CEU**	0.420	-	0.467	0.453	0.443	0.370	0.556	0.394	0.491	0.540	0.378
**GIH**	0.409	0.515	-	0.469	0.464	0.377	0.541	0.404	0.500	0.519	0.384
**CHH**	0.393	0.451	0.423	-	0.532	0.365	0.495	0.389	0.548	0.439	0.379
**JPT**	0.379	0.431	0.409	0.520	-	0.347	0.468	0.373	0.534	0.421	0.363
**LWK**	0.579	0.483	0.446	0.479	0.465	-	0.549	0.591	0.529	0.488	0.614
**MEX**	0.413	0.505	0.445	0.451	0.436	0.382	-	0.397	0.490	0.488	0.401
**MKK**	0.553	0.501	0.465	0.497	0.487	0.576	0.556	-	0.532	0.506	0.570
**CHM**	0.435	0.489	0.452	0.549	0.546	0.404	0.538	0.418	-	0.484	0.416
**TSI**	0.429	0.575	0.501	0.469	0.460	0.398	0.573	0.424	0.517	-	0.393
**YRI**	0.590	0.466	0.428	0.469	0.459	0.579	0.544	0.553	0.514	0.454	-

*The sharing proportion is calculated as the number of sharing CNVRs between population i and j divided by the number of CNVRs in population j, where i and j correspond to the population in i-th row and j-th column respectively. CHH: Chinese Han in our study combined with HapMap CHB and CHD; CHM: Chinese Minorities in our study.

#### Allele frequency of CNVs and population differentiation

Under the assumption of Hardy-Weinberg Equilibrium and a three-allele system (loss, gain and normal allele), we can use Expectation-Maximization algorithm to calculate CNV allele frequency. According to the genotypes assigned to each CNVR we identified here, 175 regions had loss allele with frequency >10% and 32 regions had gain allele with frequency >10% across 7 ethnic groups (Table S1). The allele frequency distribution indicated that loss-allele frequency was much higher than gain-allele but the overall frequency of both variants was low (Figure S5A). Even on those multi-allelic regions, loss-allele still contributed the most (Figure S5B).

**Figure 5:**
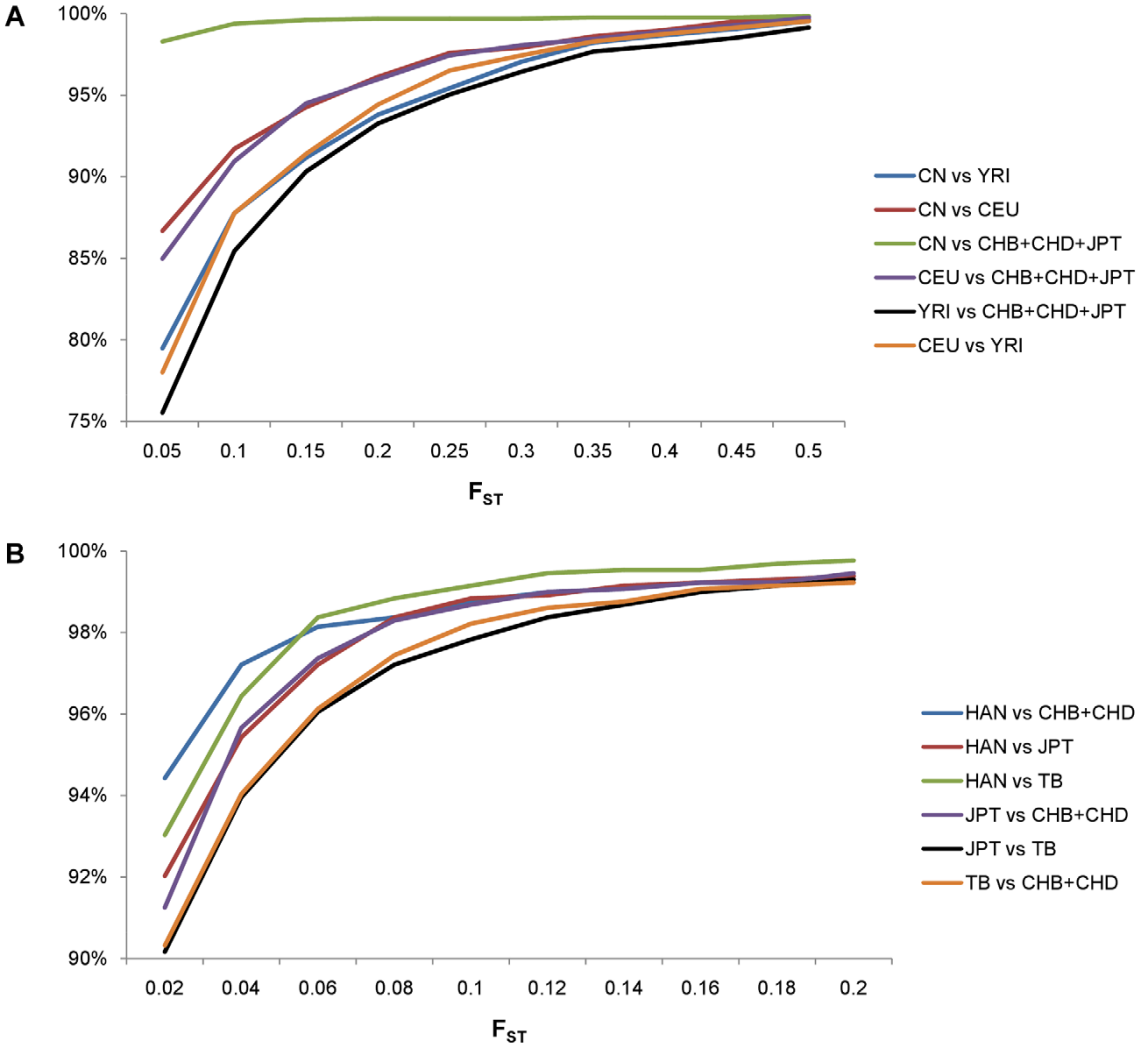
F_ST_ distribution. (A). F_ST_ distribution among world-wide populations. (B). F_ST_ distribution within Asian populations. CN: our 155 Chinese samples; TB: 41 Tibetan samples; HAN: our 80 Han Chinese samples.

Although there are many reports of detecting novel CNVs in different populations [20], [22], [31]–[34], and some of which also have assessed the extent of population differentiation based on CNV information [2], [14], [16], [18], [19], [24], to our knowledge, none of the studies included Chinese minority groups. Using CNP data, we first calculated the F_ST_ [35] between our Chinese samples (Han Chinese and Tibetans) and HapMap samples (Africans (YRI), Europeans (CEU), and Asians (CHB, CHD and JPT)). We found that F_ST_ between YRI and any other groups was larger than that between any other two populations, which was consistent with the knowledge of recent African origin of modern humans. We also expectedly observed the flat F_ST_ curve between our Chinese samples and HapMap Asian samples (Figure 5A). Furthermore, we focused on the F_ST_ distribution within Asian groups, consisting of our Han and Tibetan samples as well as HapMap Asians. In general, F_ST_ within Asian groups is much smaller than that in world-wide populations. The biggest F_ST_ was observed between Tibetans and Japanese. F_ST_ between Tibetan and Han Chinese was in the middle of that between Tibetan and Japanese and that between our Han Chinese and HapMap Han Chinese. However, F_ST_ between Tibetans and HapMap Han Chinese was slightly higher than that between Tibetans and our Han Chinese (Figure 5B), which might be due to the less complicated genetic background of HapMap Han Chinese samples compared with our Han Chinese samples.

#### Population structure based on CNV data

**Figure 6:**
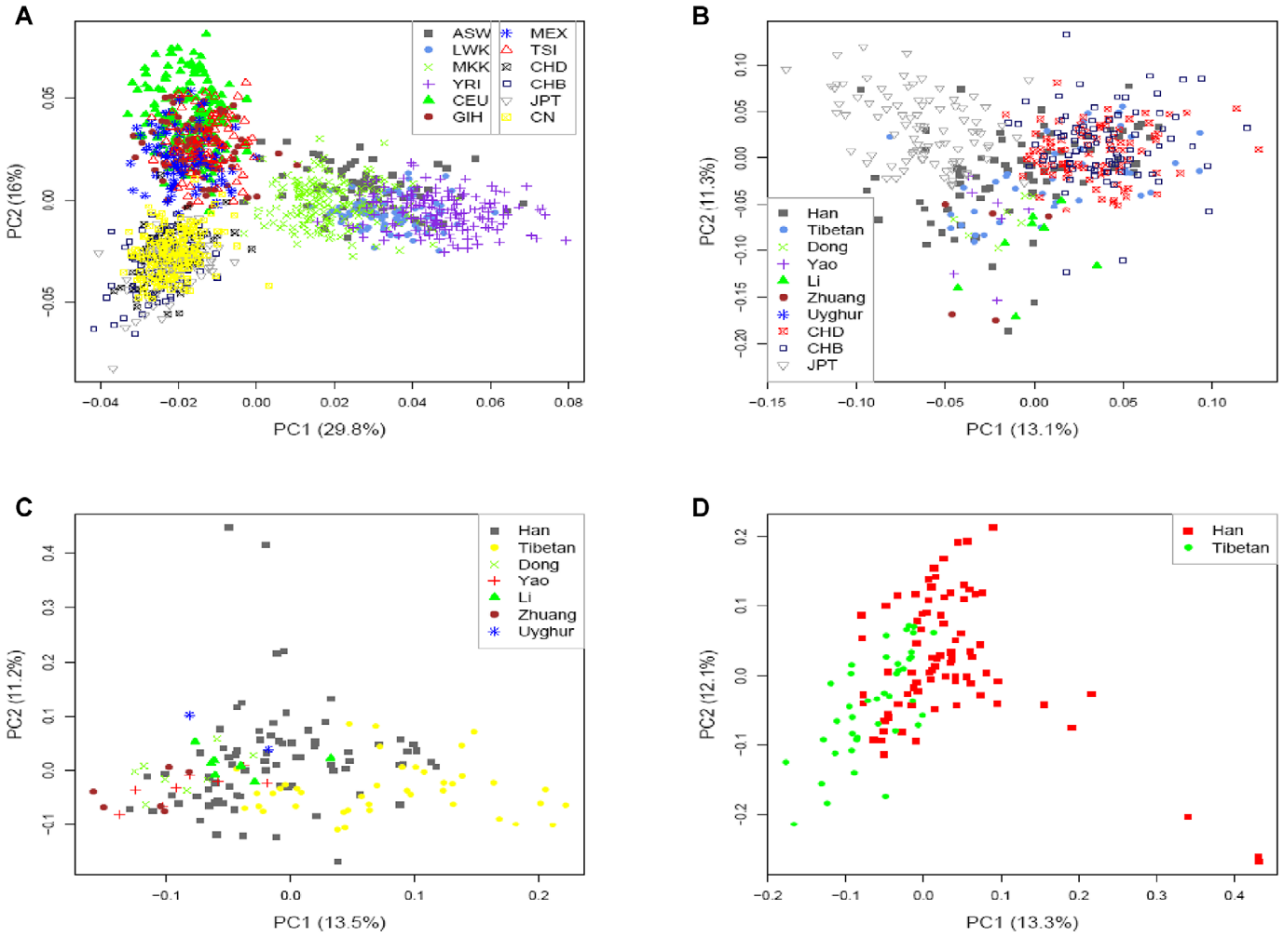
Population structure inferred by biallelic CNPs. Principal component analysis was used to detect population structure. The results were plotted as the first principal component and second principal component. (A). Our Chinese samples with all HapMap groups, 349 CNPs. (B). Our Chinese samples with HapMap Asian groups, 606 CNPs. (C). Seven ethnic groups from our Chinese samples, 643 CNPs. (D). Han Chinese and Tibetans from our Chinese samples, 600 CNPs.

Uncovering population genetic structure is both helpful in tracing the human population history [36] and crucial in unraveling the genetic basis of disease without confronting false discovery results due to population stratification [37], [38]. Many studies have produced high resolution inferences of genetic structure in various populations by SNP data [26], [39]–[41]. Although such structure has been reported by previous studies [2], [14], the spectrum of structure revealed by CNV data remains poorly understood. Here we performed a series of analysis to investigate the genetic structure of CNV hierarchically. Non-biallelic CNPs were excluded for the analysis as only allele state of biallelic CNVs could be determined at individual level. First, we took principal component analysis (PCA) on 349 biallelic CNPs from a mixture of samples, which included our 155 Chinese and all 1247 HapMap phase III samples, to test the existence of population stratification. The individuals were plotted along the axes which represent the first two principal components. The result showed evident genetic clusters – the African populations were distinguished by the first component, while European populations and Asian populations were separated by the second (Figure 6A). Then, we investigated the structure within Asian groups. The first two principal components still work in discriminating the variation inherent to Japanese. However, instead of clustering with CHB and CHD closely, our Han Chinese individuals dispersed more than HapMap Han samples, which could be due to the diverse geographic sampling locations compared with HapMap Han samples (Figure 6B). Furthermore, we tested population structure within our Chinese samples. The 643 biallelic CNP loci included in the analysis did not provide clusters as clear as those at worldwide or continent scale as they were not informative enough – many loci with high F_ST_ were multi-allelic (Figure 6C). Although it was difficult to differentiate all the ethnic groups in our Chinese samples, the result was better in the case of two ethnic groups (Han Chinese and Tibetans), in which the separation could be manually observed (Figure 6D). To sum up, population structure can be uncovered by CNVs at different scales, whereas more informative loci are required in the analysis of fine-scale inference.

#### Population specific CNVs

**Figure 7:**
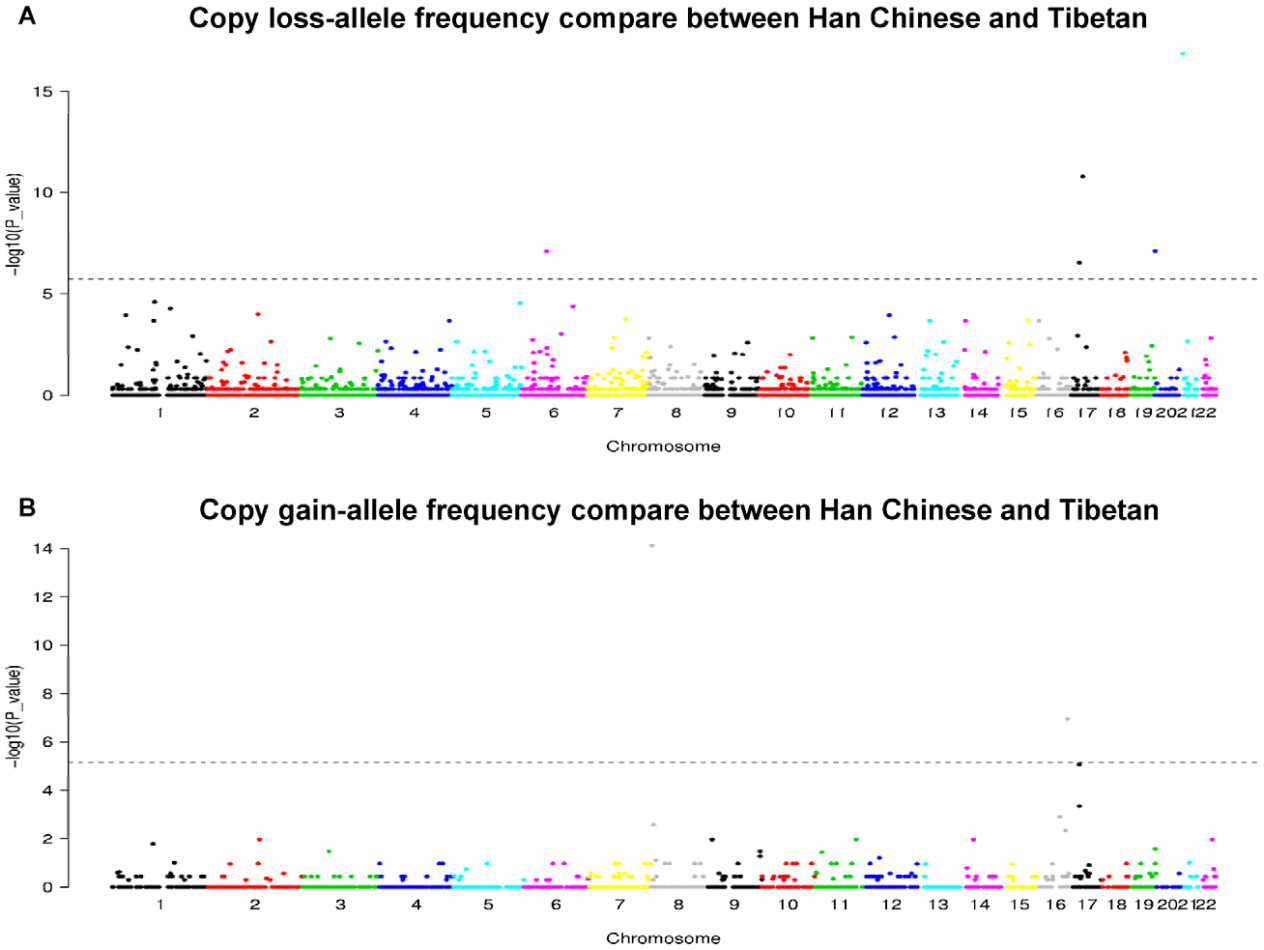
Genomic population specific CNV significance plots. The loss-allele and gain-allele frequency significance between Han Chinese and Tibetan is plotted as −log10 value in (A) and (B) respectively. Each point represents one CNV event. The dashed horizontal line indicates Bonferroni adjusted significance threshold.

Previous studies have revealed some CNV outliers of population differentiation, which suggested the existence of ethnic-specific selective pressures [2], [17], [42], [43]. In this study, however, we mainly explored ethnic-specific CNVs in Han Chinese (combined with HapMap Han Chinese samples: CHB and CHD) and Tibetans by using two different methods. On one hand, we made pairwise frequency comparison of our test group with the reference groups consisting of all the other HapMap populations one by one. The p-value was generated by testing the proportion of loss and gain against non-loss and non-gain counts, respectively. CNV loci were ranked according to the number of significant p-values in all the comparisons. The comparison between Han Chinese and Tibetans is shown in Figure 7. CNVs with highest rank of differentiation have been listed in Tables 4 and 5. For example, the top differentiated CNV between Han Chinese and other populations is at chr21:9,758,742–10,197,783, where it overlaps *BAGE* genes (B melanoma antigen) which have been found under selective pressure elsewhere [44]. Interestingly, the most significant CNV frequency difference between Tibetan and other populations with all the pairwise p-value passing the multiple comparison correction is located at chr17:31,439,592–31,653,809. In this region, a gene named *CCL3L1* was previous identified as the most differentiated gene between CEU and YRI [2], [43]. On the other hand, those CNVRs with occurrence in only one group may contribute to ethnic specific phenotype as well. However, some of those CNVRs with low frequency probably could not be captured by statistical test. Here, we focused on those non-singleton events. Using HapMap data and our Chinese samples as reference, we identified 85 Han-specific non-singleton CNVRs and 3 Tibetan-specific non-singleton CNVRs (Tables S3 and S4). We also identified ethnic-specific non-singleton CNVRs in other minority ethnic groups. Due to the relative small sample-size, we combined the other minority groups (Dong, Yao, Zhuang, Li and Uyghur), and used our Han Chinese samples as reference to search for CNVRs with occurrence only in the minority groups. There are 31 non-singleton CNVRs in our mixed minority groups that were not found in our Han Chinese samples. (Table S5). On the top occurrence of them, there is a common deletion (chr7:36,641,086–36,647,295) in Dong and Zhuang (44.4% and 50%) which overlaps two keratin-associated protein genes (*KRTAP9-3* and *KRTAP9-9*) contributing to the structure of hair fibers (RefSeq). Considering the small sample size of the ethnic groups, we cannot neglect the occurrence of CNVs on other regions, even though they are minor.

**Table 4:** Highly differentiated CNVRs between Han and other population.

CNV region			P-value (−log10) between Han and reference population											overlapping_genes
chr	start	end	TB	JPT	MEX	GIH	TSI	CEU	MKK	ASW	LWK	YRI	overall	
16	54,352,452	54,428,636	2.27	2.39	6.43	6.55	14.44	17.57	35.22	17.19	22.78	39.93	63.08	CES1,CES4
9	111,763,205	112,103,661	2.60	2.71	8.82	8.54	14.26	18.30	57.53	27.85	33.05	56.27	60.89	AKAP2,C9orf152, PALM2-AKAP2,TXN
21	9,758,742	10,197,783	7.08	15.18	19.05	11.88	12.81	4.48	12.57	10.99	18.73	10.93	25.19	BAGE,BAGE2,BAGE3,BAGE4, BAGE5,TPTE
19	59,410,750	59,446,197	2.71	3.35	4.69	6.48	10.78	17.60	16.31	20.39	30.24	36.85	21.67	LILRA6,LILRB3,LILRB5
16	72,912,614	73,023,015	11.72	6.67	4.84	5.80	4.44	5.78	13.12	11.36	3.03	3.93	2.18	CLEC18B,LOC283922

**Table 5:** Highly differentiated CNVRs between Tibetan and other populations.

CNV region			P-value (−log10) between Han and reference population											overlapping_genes
chr	start	end	HAH	JPT	MEX	GIH	TSI	CEU	MKK	ASW	LWK	YRI	overall	
17	31,439,592	31,653,809	10.77	7.58	15.23	19.37	9.04	26.55	58.01	14.27	9.96	53.65	28.53	CCL3,CCL3L1,CCL3L3, CCL4,CCL4L1,CCL4L2, TBC1D3B,TBC1D3C
7	100,167,180	100,170,778	3.74	5.48	6.54	6.94	6.87	12.08	10.48	6.74	6.94	6.27	18.92	ZAN
15	78,306,860	78,313,665	3.70	2.53	3.03	3.20	3.17	5.42	4.67	3.11	3.20	5.64	16.43	NOT_FOUND
20	1,500,116	1,547,078	7.09	3.24	2.23	7.66	6.41	4.40	28.81	10.87	13.78	20.51	14.53	SIRPB1
12	69,766,565	69,808,664	3.94	2.16	2.03	2.14	2.12	3.53	2.81	2.08	2.14	3.67	13.90	TSPAN8
19	56,817,068	56,847,282	2.44	2.22	15.89	8.21	13.40	21.55	11.59	14.32	9.12	13.11	13.35	SIGLEC14,SIGLEC5
13	78,100,453	78,107,836	2.62	2.02	3.03	2.50	3.17	5.42	4.67	3.11	2.50	5.64	11.26	RNF219

#### Linkage disequilibrium between CNPs and SNPs

**Figure 8:**
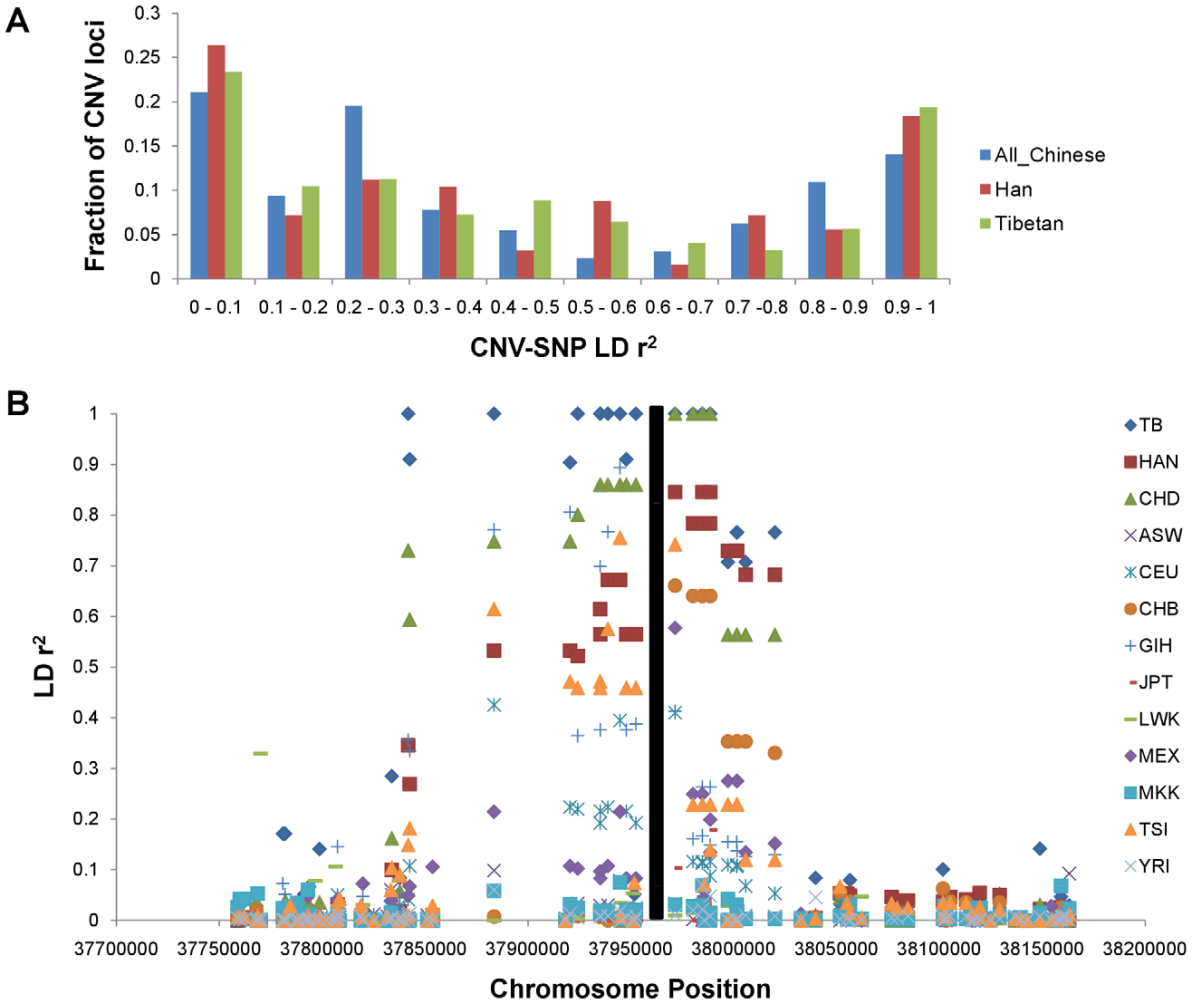
Linkage disequilibrium between CNPs and SNPs. (A). SNPs taggability of all 155 Chinese, 80 Han and 41 Tibetan for common CNV regions (frequency>10%). SNPs located within 20 kb of either ends of CNV regions were included and SNP with maximum *r^2^* among each CNVR was counted. (B). A deletion chr3:37,957,109–37,969,705 where LDs are almost perfect in Tibetan and CHD but weak in other populations. Black bar represents the location of this CNV.

Previous studies focused on the linkage disequilibrium of CNPs and SNPs in CEU and found a moderate or higher degree between them [16], [43]. Here, we tested in our Chinese populations whether CNPs can be tagged by flanking SNPs. The biallelic CNPs with events frequency larger than 10% were phased with neighboring SNPs from 20 kb on either side of CNP boundaries by cnvHap [45]. However, only 23.4% (30/128) of common CNPs in all 155 Chinese samples can be captured by at least one flanking SNP (*r^2^*> = 0.8, Figure 8A), which is much lower than that in previous studies reporting CEU [16], [43]. The number of flanking SNPs is likely to account for the loss of taggability, because the ascertainment bias of both SNP discovery and array design exit between Asian and European populations, and we found that the average number (14.7) of flanking SNPs for high ‘taggability’ CNPs (*r^2^*> = 0.8) was marginally significant than that (11.7) of low taggability CNPs (*r^2^*<0.8). (P-value = 0.0509, two tails t-test), which indicates that further efforts should be made on array design if we want to fully understand CNV characteristics in Chinese populations.

Due to the limited sample size, we mainly tested LD in Tibetan and Han Chinese for ethnic groups. Both of the two groups have similar LD pattern of high ‘taggability’ CNPs (*r^2^*> = 0.8, Figure 8A). However, Tibetan had over 50% of perfect tag regions (*r^2^* = 1) than Han Chinese. Interestingly, by manually checking pairwise difference of *r^2^* of each SNP between Tibetan and Han Chinese, we found a common deletion (chr3:37,957,108–37,969,705; Tibetan frequency 24.4%) where the flanking SNPs were in strong LD with this CNV in Tibetan but not in Han Chinese. Whereas this deletion was common in other HapMap populations, none of them had such strong SNP LDs except CHD (Figure 8B). Although it is not clear whether selection forces have shaped the LD pattern of this region, given that both Tibetan and CHD live on highlands, the only known gene named *CTDSPL* overlapping with this region is worth of further study.

## Discussion

The widespread copy number variation in the human genome greatly contributes to human phenotypic divergence and influences predisposition to disease [7], [46]–[48]. By using Affymetrix SNP 6.0, we have generated a comprehensive CNV map of Chinese by analyzing 155 healthy individuals coming from 7 ethnic groups. Population analysis based on CNVs of individuals from diverse ethnic groups reveals the differentiation and uncovers the genomic CNV distribution as well as population structure at different levels. In addition, we have identified -population-specific CNVs from Han Chinese, Tibetans and other ethnic groups which could be candidate loci under positive selection.

In our study, the number of deletion count is much more than that of duplication while the size of duplication is much larger than that of deletion. Such patterns were also observed in other studies using different chips and algorithms [17], [49]. Another comprehensive CNV study using tiling oligonucleotide microarrays being comprised of 42 million probes observed a 5∶1 ratio of deletions to duplications as well [43]. Moreover, we made a comparison between HapMap individual CNVs analyzed by Affymetrix SNP 6.0 array and Agilent 105K CNV genotyping array [43]. Excluding CNV loci that were not genotyped by Agilent 105K CNV genotyping array, the overall validation rate (CNV detected by both Affymetrix SNP 6.0 and Agilent 105K) of more than 400 HapMap individuals was 0.768 and the consistent genotype proportion based on those validated events between two methods was 0.927. Considering that the validation rate was based on two totally different platforms and inferred by two different algorithms, such consistency is shown to be satisfying and indicates the accuracy of our whole analysis.

We constructed 1,440 CNV regions in our Chinese samples, in which 170 (23.2%) singleton CNVs and 5 (0.7%) non-singleton CNV regions were not found in previous studies submitted to DGV or other HapMap populations. However, these numbers might be underestimated due to large boundaries reported by previous studies using low-resolution platforms [16], [29]. Considering our relatively small sample size here, it is hard to distinguish whether these large proportion of singleton CNVs are true ‘rare events’ or not, indicating that CNV detection in Chinese population, being totally comprised of 56 ethnic groups, is far from complete.

CNVR is a widely used term to represent the union of overlapping CNVs at a particular region and measures the cumulative extent of copy number variant [2], [6], [15], [17]. Although it provides the general knowledge of copy number variation on a given genomic region, CNVR itself may not be enough to capture all the information of some complex regions due to the inconsistent CNV boundaries across different individuals. Thus, we applied ‘CNVseg’ to obtain the full information by partitioning CNVRs into indivisible segments according to the breakpoints of each CNV. These non-overlapping segments would be helpful while carrying out fine-scale analysis, for instance, retrieving the sub CNV regions in a given CNVR and mapping disease associated genetic elements. We also observed a small portion of CNVRs (17 out of 708 non-singleton CNVRs) where at least one individual present both deletion and duplication, of which five regions seem to be comprised of more than one sub regions. CNVs on these regions should be analyzed with caution. Having noted that factors such as signal to noise ratio and sensitivity of calling algorithm etc. would influence the results of CNVs, we suggest including both CNVR and CNVseg in performing CNV association analysis.

Whereas some allelic states of CNV cannot be determined, their allele frequencies are attainable. We inferred allele frequency by using the EM algorithm based on two major assumptions - ‘three allele system’ and Hardy Weinberg Equilibrium (HWE). The first assumption holds true as long as the genotype varies from 0-copy to 4-copy, which stands for most CNP loci (99%, 1279/1291) according to Birdsuite, version 1.5.5 [25]. The violation of second assumption could occur, for instance, when some CNVs under selection pressure. However, previous study found that the deviations from HWE did not significantly affect SNP haplotype inference and impacted on the methods using the EM algorithm minutely [50], [51]. Hence, it is feasible to use EM algorithm to infer allele frequency for most CNVs. Based on the allele frequency of CNP, we used F_ST_ to measure the differentiation between populations. Generally, the results of F_ST_ distribution accord to our knowledge and the diverse sample sites in our Han Chinese might account for the slight deviation in the F_ST_ distribution. However, the complexity of Han Chinese genetic background in essential should not be ignored when conducting disease studies.

The genetic distance devised in this study has been proven to be capable of recovering the genetic relationship among Chinese ethnic groups, which, to some extent, reflects a linguistic pattern. Previous study using other genetic data has suggested that Y-chromosome haplogroups correlates with linguistic classification [52], however, whether such pattern exists in CNV data requires further study with larger sample size and inclusion of more ethnic groups. While different ethnic groups have various levels of CNV diversity, they share a certain amount of CNV loci. For example, our CNV sharing analysis points out that the sharing by at least two ethnic groups within our Chinese samples is up to 80% of all the non-singleton CNVRs. Even at world-wide scale, the sharing by at least two continent groups is still more than 50%. However, this finding is contrary to the result of a study which focused on HapMap CEU and YRI using a designed array to detect CNVs. They found that the proportions of overlap CNV regions between two populations were only 25% and 17% for CEU and YRI, respectively [53], but our pairwise sharing analysis showed that the corresponding numbers are 46.6% and 37.8%. This discrepancy could be due to CNV detecting platforms or the large portion of rare events they observed.

We explored the population structure of our Chinese samples at different scales by using PCA of biallelic CNP. The population genetic structure of CNVs does exist at various levels and can be inferred at certain scale by biallelic CNVs; however, the power of discriminating variants inherent to each ethnic group decreased as the analysis scale gets narrowed. The differentiation of each ethnic group in our Chinese samples could not be easily distinguished when all of our ethnic groups were involved. This is due to the paucity of informative loci in biallelic CNPs. Most of the multi-allelic CNPs excluded are more informative; for example, multi-allelic CNPs with F_ST_ >0.01 comprised ∼40% (42/106) of the total CNPs with F_ST_>0.01. Given such a big proportion of information loss, the poor power of inferring population structure by biallelic CNVs at minute-difference scale is predictable. Considering the success of applying high-density SNPs as markers to detect population structure, it is unwise to use CNV data for structure inference. However, what is interesting is to compare the patterns inferred by CNV and SNP when they have comparable information.

There are several signals highlighted as we compare CNV frequency difference among populations. For example, *CCL3L1*, which was identified as the most frequency differentiated CNV overlapping gene between CEU and YRI [2], [43], showed also the most significantly differentiation between Tibetan and other populations. Interestingly, GIH was a most extreme case that there was no copy loss found at this CNV locus. Except GIH, Tibetan had the least copy loss proportion which was just about 20%, while for the rest populations compared in this study, the loss proportions were all higher than 50%. Besides, there were also some loci which Han Chinese and Tibetan shared a similar frequency spectrum and differed from other populations (Tables S3 and S4). Moreover, as a complementary of population specific CNV analysis, it is identified that those non-singleton CNVs only occurred in one population, which were more likely ‘buried’ in the frequency comparisons but was probably important as well in contributing to phenotypic diversity. Among the five novel non-singleton CNVRs (neither reported in DGV nor discovered in HapMap samples) identified from our Chinese samples, two regions (chr16: 7,130,898 – 7,174,339 and chr21:43,188,383 – 43,194,988) with loss events detected solely in Tibetan and Han Chinese, respectively are both overlapping with genes. According to RefSeq, human gene ataxin 2-binding protein 1 isoform 4(*A2BP1*) - the one overlapped with Tibetan specific loss region may contribute to the restricted pathology of spinocerebellar ataxia type 2 (*SCA2*). Ataxin-2 is the gene product of the *SCA2* gene which causes familial neurodegenerative diseases. The other interesting gene overlapped with Han Chinese specific loss region is human gene NADH-ubiquinone oxidoreductase flavoprotein 3 (*NDUFV3*). The protein produced by this gene a subunit of the NADH-ubiquinone oxidoreductase complex which is part of the mitochondrial respiratory chain and catalyzes the rotenone-sensitive oxidation of NADH and the reduction of ubiquinone.

As members of a big Chinese family, the genetic contribution of other minority ethnic groups (Dong, Yao, Zhuang, Li and Uyghur) cannot be ignored. Considering the small sample size of each minority group, we combined them as one and compared it with Han Chinese by searching for non-singleton specific regions. Thirty-one regions were identified, of which 40% overlap with genes. Although these CNVs did not occur so frequently, given the limited sample size we included in this study, some of them were probably common in each specific group. All these differentiated CNVs that might play a role in phenotypic differences among human populations are interesting candidates for future studies. Accordingly, the diversity of Chinese remains to be discovered and the large sample size of ethnic groups will be needed in future genomic variant analysis.

In our study, SNP taggability of CNPs in Han Chinese and Tibetan population is not as high as that in previous studies reported in CEU [16], [43]. Such missing is possibly due to ascertainment bias of array design. By further comparing the LD between populations, we found an interesting region which shows potential population specific signal. This region overlaps a gene named *CTDSPL* (*RBSP3*) which is a member of small C-terminal domain phosphatases gene family possibly controlling the RNA polymerase II transcription machinery [54]. It is ubiquitously expressed in lung and other normal tissues and characterized as a tumor suppressor gene [54], [55]. However, such signals might be overlooked if we only consider CNVs or SNPs separately. Joint analysis of different variants will improve the power of analyzing population diversity.

Our results provide a comprehensive CNV map of Chinese from seven ethnic groups. At population level, we analyzed CNV genomic distribution and diversity which indicate the genetic relationship among human populations and suggest a linguistic pattern within Chinese ethnic groups. Different populations share a large percentage of CNV regions: the closer the relationship, the higher the sharing. We also demonstrated that the impacts of CNVs among populations could be reflected by detecting population structure as well. Our joint analyzing results highlighted those most differentiated CNVs among Chinese ethnic groups which are candidates to be further studied about their functional consequences as they are more likely under selective pressure than the others. In summary, our results present here will serve as a Chinese CNV resource to assist characterizing the pattern of human genetic variation, performing population level analyses and investigating medical disorders.

## Methods

### Sample collection and SNP genotyping

There are 184 samples in total, collected from 7 Chinese populations representing major linguistic groups in China, including 101 Han Chinese, 46 Tibetans, 9 Dongs, 8 Yaos, 8 Zhuangs, 8 Lis, 4 Uyghurs. All samples were assayed on the Affymetrix Genome-Wide Human SNP Array 6.0 which contains both SNP probes and copy number probes for the simultaneous detection of SNPs and CNVs [16]. Quality control for SNP calling was performed by apt-geno-qc from Affymetrix Power Tools. Samples with call-rate >0.86 were considered to pass the first quality control. This step excluded 14 Han Chinese, 2 Tibetans and 2 Zhuang Chinese from downstream analysis.

Samples which were also assayed by Affymetrix Genome-Wide Human SNP Array 6.0 from the International HapMap Project [56] have been downloaded from website and included in some population analyses. The raw intensity data of these samples were analyzed with Birdsuite, version 1.5.2 [25] as well and followed the same quality control criterions as our data. Finally these HapMap samples consist of 86 ASW (African ancestry in Southwest USA), 167 CEU (Utah residents with Northern and Western European ancestry), 89 CHB (Han Chinese in Beijing, China), 90 CHD (Chinese in Metropolitan Denver, Colorado), 89 GIH (Gujarati Indians in Houston, Texas), 90 JPT (Japanese in Tokyo, Japan), 89 LWK (Luhya in Webuye, Kenya), 83 MEX (Mexican ancestry in Los Angeles, California), 177 MKK (Maasai in Kinyawa, Kenya), 88 TSI (Toscans in Italy) and 175 YRI (Yoruba in Ibadan, Nigeria). According to the results of population structure inferred by principal component analysis using SNPs (data not shown), we classified ASW, LWK, MKK and YRI as African population; CHB, CHD and JPT as Asian population; CEU, GIH, MEX and TSI as European population.

### CNV detection

CNV detection was performed by Birdsuite, version 1.5.2 [25]. Birdseye, one of the packages in Birdsuite, is a Hidden Markov Model based algorithm and is used to detect novel CNVs in each sample. According to the results of Birdseye, we filtered segment with length less than 1 kb or number of makers less than 3 or LOD score (which describes the likelihood of the segment of being a current state calling relative to other copy state over a given region) less than 5. Then, we summed up the CNV events called by Birdseye for each individual and manually removed the individual with extreme large number of CNV events, which were most likely due to the large noise of sample. The remaining samples with excessive number of either deletion or duplication events (>5 s.d. above the mean number of CNVs per individual) were also excluded from downstream analyses. In addition, 5 replicated samples (all from Tibetan group) with relatively lower SNP call rate were removed. After the CNV quality control at sample level, there are 155 samples included in the CNV analyses, which are comprised of 80 Han Chinese, 41 Tibetans, 9 Dongs, 8 Yaos, 6 Zhuangs, 8 Li s, and 3 Uyghurs.

The genotypes of 1,315 copy number polymorphisms (CNP) which are defined by a previous study of HapMap samples [16] can be determined by another package Canary of Birdsuite. Samples passed the preliminary quality control were subjected to additional filtering steps: those copy number genotype callings with uncertainty showing large confidence sore (>0.1) were treated as missing data, and we chose the callings with higher LOD scores as the final copy state in case some of the callings in the same genomic locations are inconsistent between Birdseye and Canary. The combined results were used to construct the CNV map and further population genetic analysis.

Only the results of autosomes were included in the analyses because CNV callings of the sex chromosomes are probably not as reliable as those of autosomes due to the segmenting problem with both X and Y chromosomes. Moreover, a great portion of large CNV events (>1 Mb) was analyzed separately from those CNVs less than 1 Mb long, because they could be artifacts, for example, due to the scarcity of probes near the centromeres of the chromosome. The workflow of detecting and filtering CNV is shown in Figure S1.

### CNV population analysis

#### Construction of CNV map

All probe coordinates were mapped to the human genome assembly build 36 (hg18). Because of the platform limitation of SNP array in determining CNV boundaries, they could only be approximated by the first and the last probe positions. In order to construct the comprehensive autosomal CNV map in Chinese population, we apply the widely-used term Copy Number Variable Region (CNVR) to delineate the characteristics. A CNVR refers to a union region of overlapping CNVs on the chromosome [6]. Here we adopt the definition in a previous study [49], that is, to merge CNVs from different samples with any amount of overlap by extending the boundaries of the overlapping CNVs. We combined both Canary and Birdseye results to generate CNVR map. Gene ontology of the genes overlapping with this map was performed to measure the enrichment of these genes compared with the rest genes of the human genome by Gene Ontology Tree Machine [30].

In order to calculate the allele frequency and carry out population analysis, we assigned each individual a ‘genotype’ for each CNVR by the following criteria: if no CNV was found in that CNVR of the individual, we assign the calling as normal state (which is assumed as ‘two’); if one was found, then the genotype is the CNV state; if more than one CNV were found in that CNVR of the same individual, the genotype of that CNVR is assigned by the copy state of CNV with the largest length in that CNVR (Figure 1A). Although such assignments might be biased, especially for some complex CNVRs (those that simultaneously have both deletion and duplication in one CNVR of one individual; Figure 1B), the genotypes can reflect the ‘true-state’ for most CNV regions due to the small proportion of the complex CNVRs (17/1440 CNVRs). One way to exclude this inconsistency is to dissect CNVR into small segments by cutting the two breakpoints of each CNV in the CNVR as described by one previous study as ‘CNV block’ [17]. Here we call such small units ‘CNVseg’ (Figure 1A). The genotype of CNVsegs can easily be determined as the copy state of the segment in that region without introducing any inconsistency. Such dissection can solve the problem of assigning genotype to a complex CNVR and serve as a complement to provide missing information of a CNVR.

#### CNV diversity and distribution pattern

We proposed a measure to characterize the genetic difference of CNV genotypes between two individuals for all the CNV regions. Distance between two populations is defined as the average of pairwise genotype differences between two individuals from the two populations respectively. Based on this, we constructed a phylogenetic tree by Neighbor-joining and UPGMA with 1,000 bootstrap replications. The consensus phylogenetic tree was constructed using PHYLIP [57].

CNV sharing analysis was conducted by counting the number of sharing regions among populations. However, the larger the sample size of the population, the more CNV regions will be generated (including singletons). The results of sharing analysis would be biased if we do not consider the affect of sample size. Therefore, to make the analysis comparable, we specified the smallest sample size of compared populations as ours. For those whose sample size were larger than this, we randomly sampled the same number of individuals and averaged 100 random sampling results.

#### Population differentiation and population structure

The calling results of CNVs by Canary and Birdseye contained five copy states: 0-copy state (homozygous deletion), 1-copy state (heterozygous deletion), 2-copy state (normal state/copy-neutral with LOH), 3-copy state (single copy duplication) and 4-copy state (double copy duplication). Such five copy state results can be explained by a three-allele (0 copy-allele, loss-allele; 1 copy-allele, normal-allele; 2 copy-allele, gain-allele) system. Based on the three-allele system, we applied Expectation-Maximization (EM) algorithm to calculate the allele frequency under the assumption of Hardy-Weinberg Equilibrium (HWE), which allows us to obtain the widely-used statistic F_ST_ [35] into characterizing genetic distance between populations in a given CNVR. As most of the CNV difference between two individuals arises from CNP and it is comparable to conduct analysis at predefined loci, we used CNP data to calculate F_ST_ and analyzed together with HapMap samples.

Population structure was inferred by principal component analysis (PCA). Due to technology limitations, allelic copy number states were only available for biallelic CNVs. Therefore, we used CNVs observed as biallelic in the analysis of populations. In order to combine the results with HapMap samples, we focused on CNPs as we did in F_ST_ calculation. We coded biallelic CNPs with genotype ‘0, 1, 2, 3, 4’ as ‘0/0, 0/1, 1/1, 1/2, 2/2’ for the pair of two chromosomes. PCA was performed by software Eigenanalysis 2.0 [58] at four different levels.

#### Population specific CNV analysis and Linkage disequilibrium analysis

We identified population specific CNVs using two complementary strategies. Firstly, we made pairwise frequency comparison one by one of our test group with the reference groups that consist of all other HapMap populations. The p-value was generated by testing on a contingency table with two populations and two types of allele counts (loss-allele and non-loss-allele or gain-allele and non-gain-allele). CNV loci were ranked according to the number of significant p-values in all the comparisons. Secondly, some of the low-frequency CNVs with occurrence only in one population might not be captured by the frequency comparison. Thus, we conducted a search for those non-singleton CNVs observed only in one ethnic group.

Linkage disequilibrium analysis was performed in biallelic CNVRs. Biallelic CNVRs with frequency larger than 10% and SNPs within 20 kb of both ends of CNVRs were phased into haplotype by cnvHap [45]. Since the sample size of some Chinese ethnic groups was small, LD was calculated as *r^2^* mainly for 155 Chinese, 80 Han Chinese and 41 Tibetan, respectively.

## Supporting Information

Figure S1 **CNV detection flow chart.** See the detailed description of the filtering procedure in Methods.
(PDF)

Figure S2 **CNV length distribution.** While the number of deletion (18,306) is more than 5 times of duplication (3,534), duplication has much larger length (median 49,801bp) than deletion (8,823bp).
(PDF)

Figure S3 **Genomic distribution of CNVs in Chinese population.** Red and blue triangles indicate the chromosomal location of deletions and duplications respectively.
(PDF)

Figure S4 **Phylogenetic tree of Chinese ethnic groups constructed by Neighbor-joining.** Phylogenetic tree of Chinese ethnic groups based on average pairwise genetic population distance between ethnic groups with 1,000 bootstrap replications by Neighbor-joining.
(PDF)

Figure S5 **Allele frequency distribution in Chinese population.** (A). Cumulative allele frequency distribution of Chinese population in 1440 CNVRs. (B). Allele frequency distribution of Chinese population in 254 multi-allelic CNVRs.
(PDF)

Figure S6 **The state of Individual CNVs on non-singleton CNVRs (CNVR length < 100 kb).** Black bars denote CNVR, and below which each bar denote a CNV call in one individual. Red and blue represent deletion and duplication, respectively.
(PDF)

Figure S7 **The state of Individual CNVs on non-singleton CNVRs (CNVR length> = 100 kb).** Black bars denote CNVR, and below which each bar denote a CNV call in one individual. Red and blue represent deletion and duplication, respectively.
(PDF)

Figure S8 **CNV sharing.** Venn diagram of (A).Deletion, (C).Duplication and (E).Multi-allelic CNVs sharing results among African, Asian and European groups (each group with sample-size 425). Venn diagram of (B).Deletion, (D).Duplication and (F).Multi-allelic CNVs sharing results among Han Chinese, Chinese minority and Japanese groups (each group with sample-size 75).
(PDF)

Table S1 **A map of Chinese Copy Number Variable Regions (CNVRs) and Copy Number Variable Segments (CNVsegs) with Allele frequency and overlapping genes including overlapping OMIM genes.**
(XLS)

Table S2 **Gene ontology analysis of CNVR overlapping genes.** According to the type of variants, CNVRs have been divided into deletion, duplication and multi-allelic groups.
(XLS)

Table S3 **Han Chinese specific CNV regions and highly differentiated CNV regions between Han Chinese and other populations.**
(XLS)

Table S4 **Tibetan specific CNV regions and highly differentiated CNV regions between Tibetan and other populations.**
(XLS)

Table S5 **Chinese Minority (Dong, Yao, Zhuang, Li and Uyghur) Specific non-singleton CNV regions compared with Han Chinese.**
(XLS)
