# Supplementary material for: A Map of Copy Number Variations in Chinese Populations
Source: PLoS One. 2011 Nov 7;6(11):e27341. doi: 10.1371/journal.pone.0027341 (PMC3210162; doi:10.1371/journal.pone.0027341)
Supplement: Figure S1 — CNV detection flow chart. See the detailed description of the filtering procedure in Methods. (PDF) [file pone.0027341.s001.pdf]

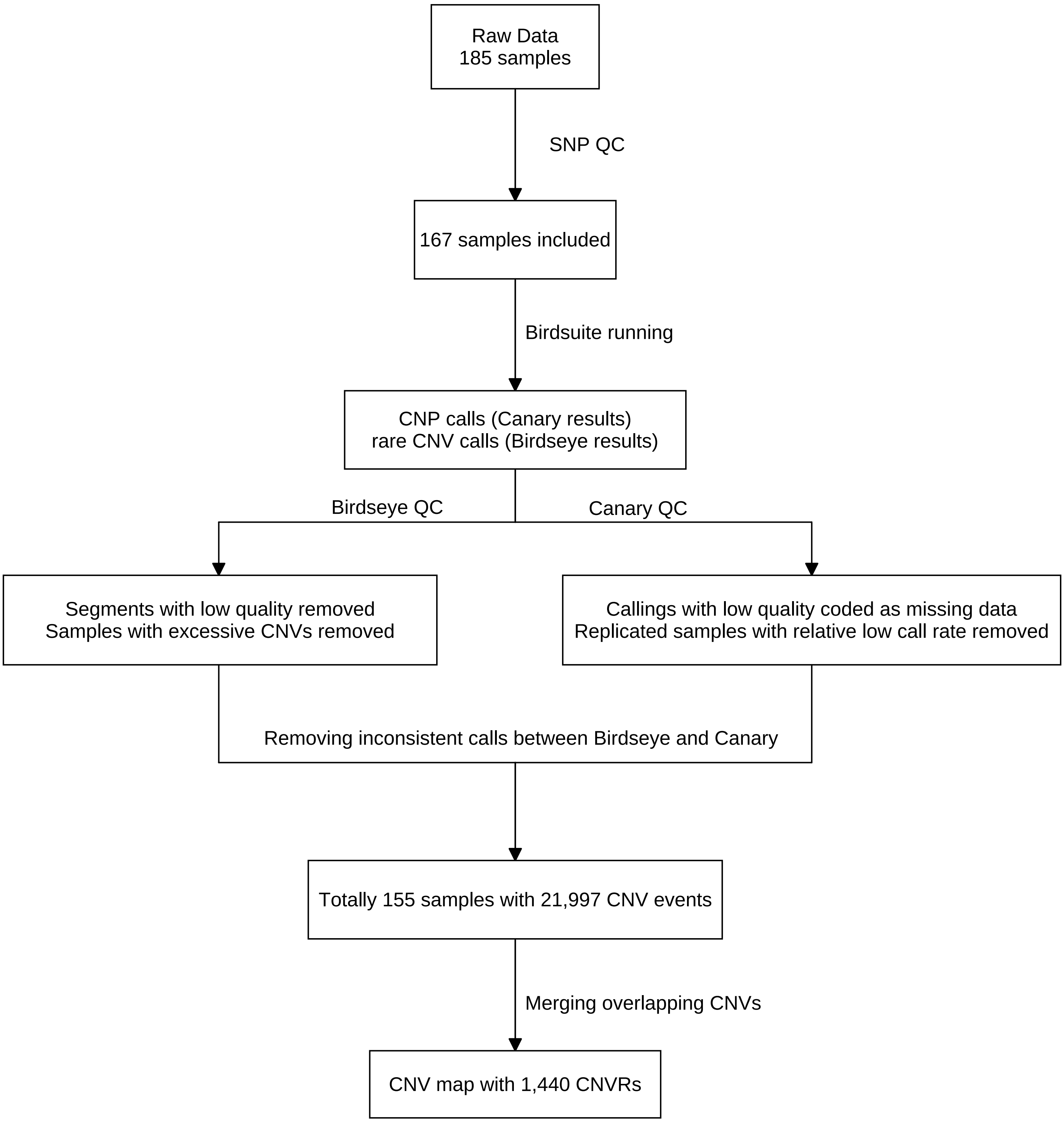

QC filter		
SNP	Canary	Birdseye
The samples with calling rate <0.86 were removed after running apt-geno-qc	Callings with confidence >=0.1 were removed	Segments which did not meet the following criterias were removed:<br>LOD score>=5<br>number of markers >=3<br>size <1,000 bp
