# Supplementary figures and images for: A Map of Copy Number Variations in Chinese Populations
Source: PLoS One. 2011 Nov 7;6(11):e27341. doi: 10.1371/journal.pone.0027341 (PMC3210162; doi:10.1371/journal.pone.0027341)

## CNV length distribution

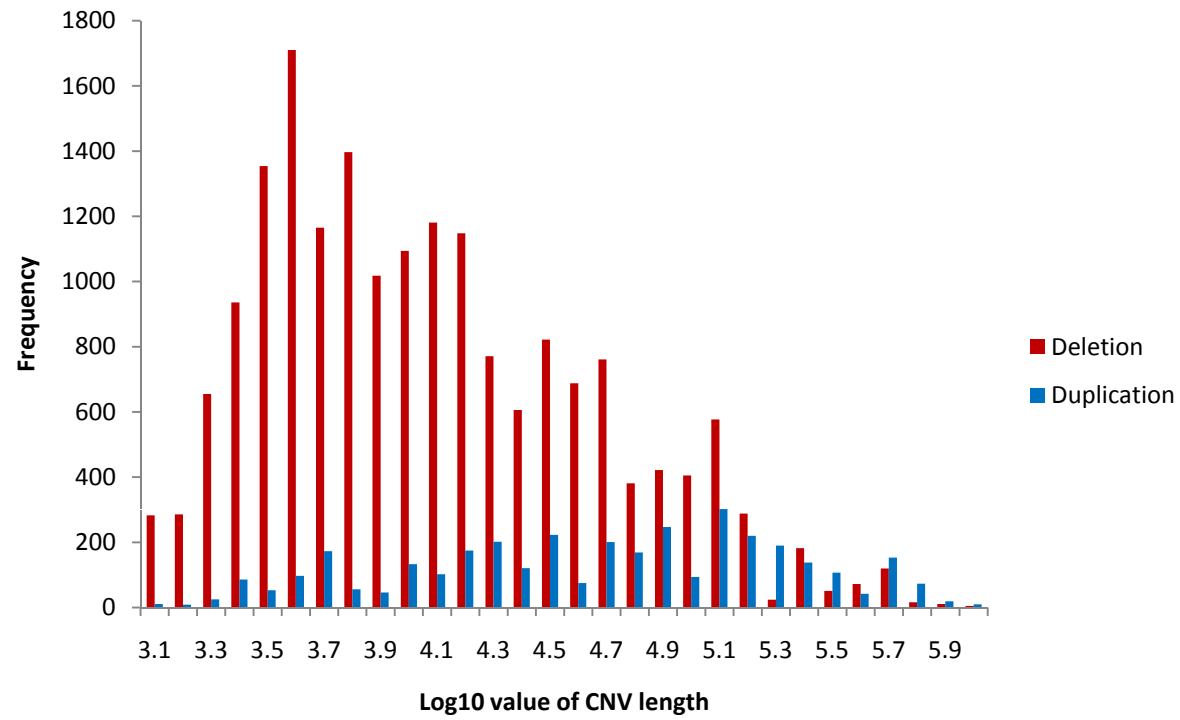

Supplement: Figure S2 — CNV length distribution. While the number of deletion (18,306) is more than 5 times of duplication (3,534), duplication has much larger length (median 49,801bp) than deletion (8,823bp). (PDF) [file pone.0027341.s002.pdf]

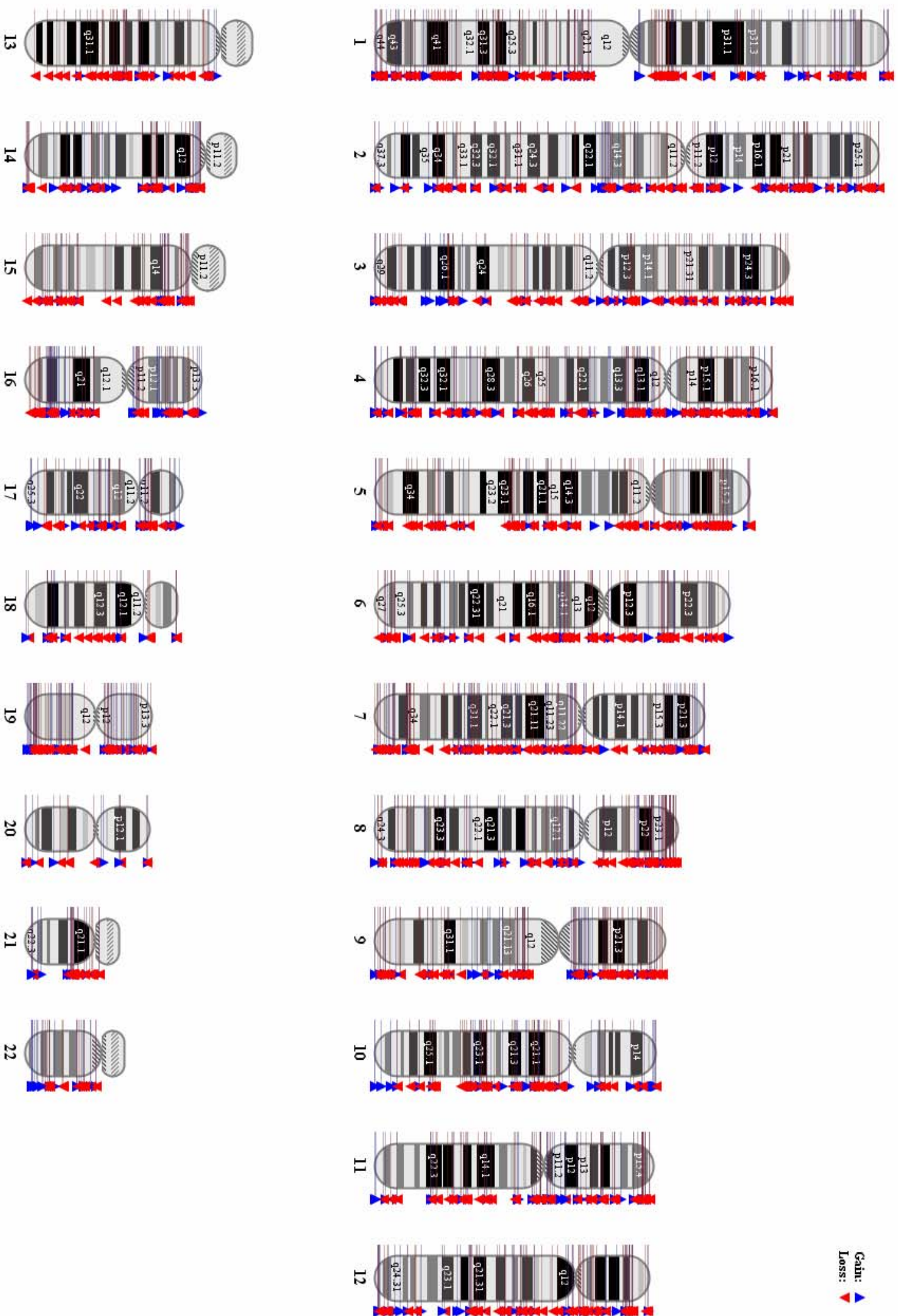

Supplement: Figure S3 — Genomic distribution of CNVs in Chinese population. Red and blue triangles indicate the chromosomal location of deletions and duplications respectively. (PDF) [file pone.0027341.s003.pdf]

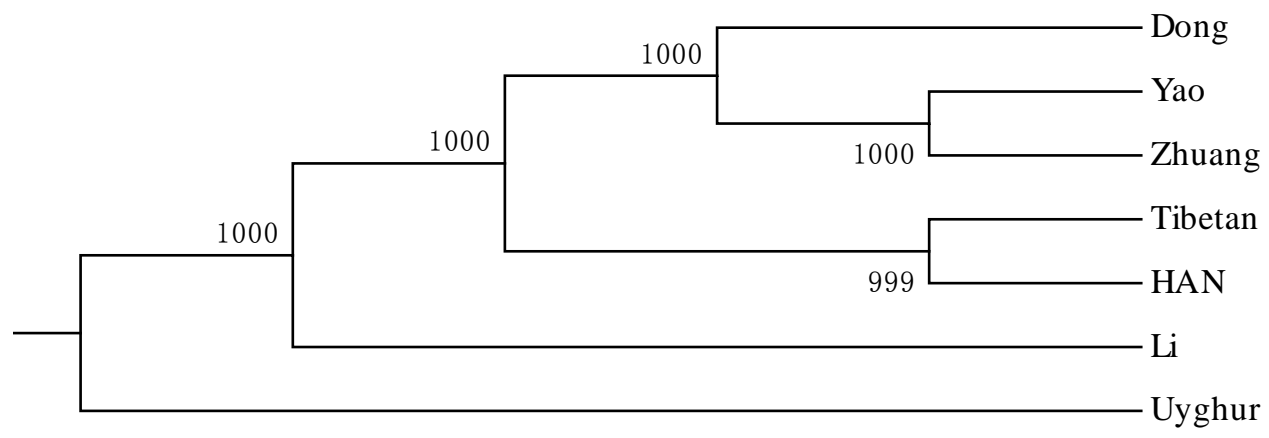

Supplement: Figure S4 — Phylogenetic tree of Chinese ethnic groups constructed by Neighbor-joining. Phylogenetic tree of Chinese ethnic groups based on average pairwise genetic population distance between ethnic groups with 1,000 bootstrap replications by Neighbor-joining. (PDF) [file pone.0027341.s004.pdf]
