# Supplementary material for: A Map of Copy Number Variations in Chinese Populations
Source: PLoS One. 2011 Nov 7;6(11):e27341. doi: 10.1371/journal.pone.0027341 (PMC3210162; doi:10.1371/journal.pone.0027341)
Supplement: Figure S5 — Allele frequency distribution in Chinese population. (A). Cumulative allele frequency distribution of Chinese population in 1440 CNVRs. (B). Allele frequency distribution of Chinese population in 254 multi-allelic CNVRs. (PDF) [file pone.0027341.s005.pdf]

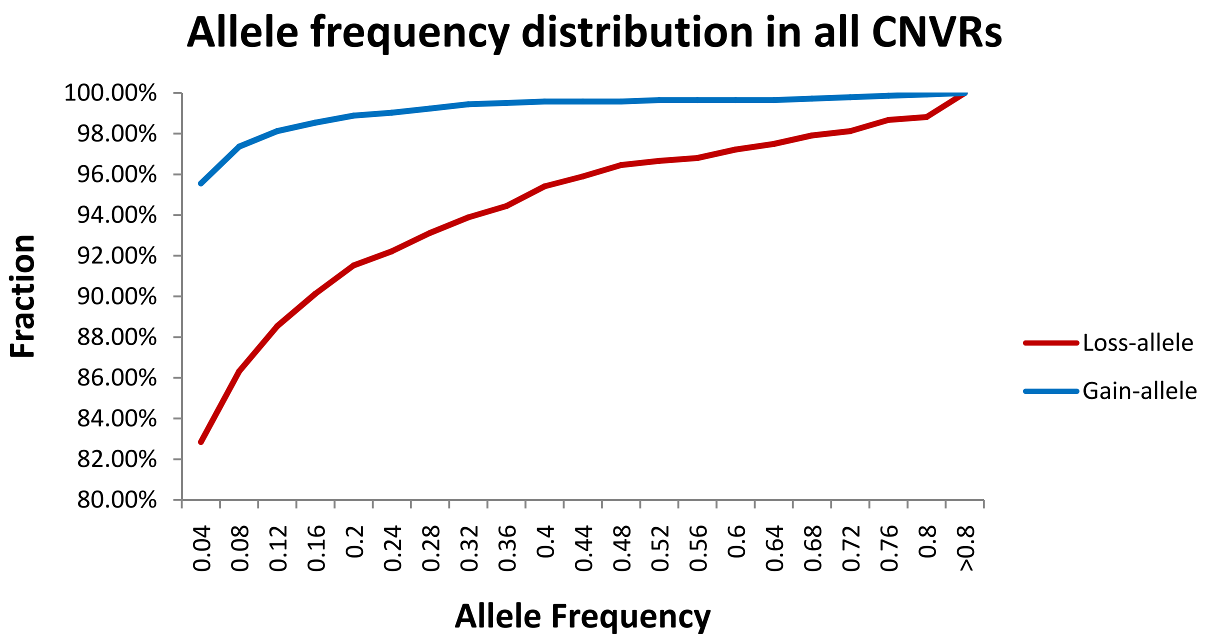

Allele frequency distribution in all CNVRs
Fraction
100.00%
98.00%
96.00%
94.00%
92.00%
90.00%
88.00%
86.00%
84.00%
82.00%
80.00%
0.04
0.08
0.12
0.16
0.2
0.24
0.28
0.32
0.36
0.4
0.44
0.48
0.52
0.56
0.6
0.64
0.68
0.72
0.76
0.8
>0.8
Allele Frequency
Loss-allele
Gain-allele

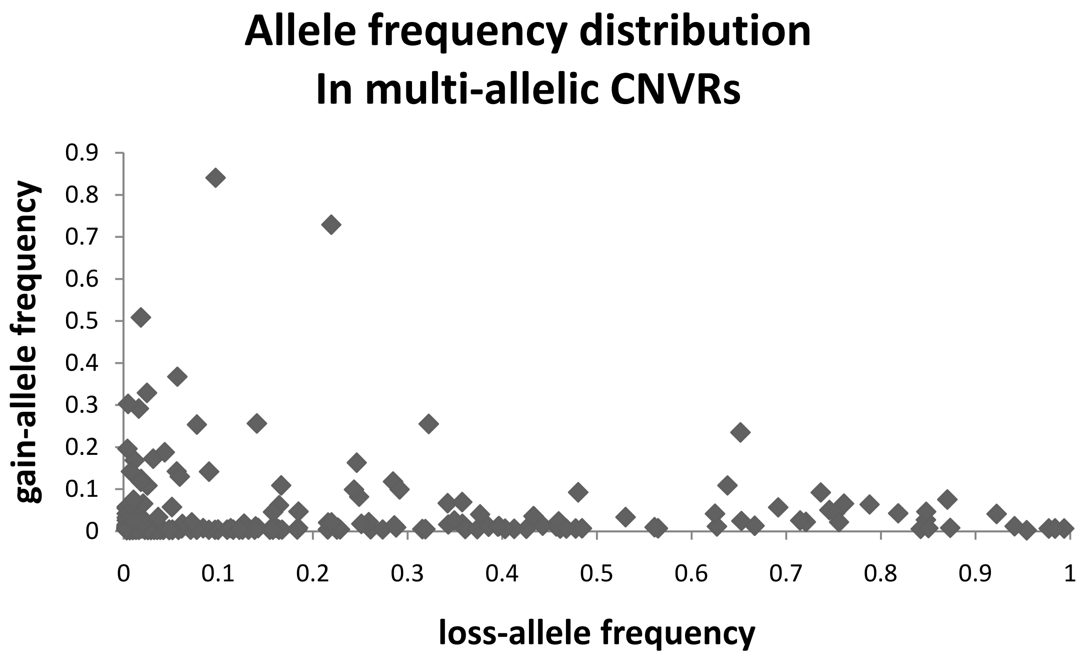

Allele frequency distribution
In multi-allelic CNVRs
gain-allele frequency
0.9
0.8
0.7
0.6
0.5
0.4
0.3
0.2
0.1
0
0
0.1
0.2
0.3
0.4
0.5
0.6
0.7
0.8
0.9
1
loss-allele frequency
