# Supplementary material for: A Map of Copy Number Variations in Chinese Populations
Source: PLoS One. 2011 Nov 7;6(11):e27341. doi: 10.1371/journal.pone.0027341 (PMC3210162; doi:10.1371/journal.pone.0027341)
Supplement: Figure S6 — The state of Individual CNVs on non-singleton CNVRs (CNVR length < 100 kb). Black bars denote CNVR, and below which each bar denote a CNV call in one individual. Red and blue represent deletion and duplication, respectively. (PDF) [file pone.0027341.s006.pdf]

chr1:51598-88465

chr1:15665011-15683808

chr1:22190770-22216789

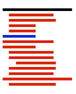

chr1:34875241-34877078

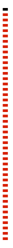

chr1:40731169-40742251

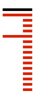

chr1:41119794-41149087

chr1:47486248-47501675

chr1:61886594-61890775

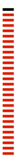

chr1:64615315-64624133

chr1:72528701-72583736

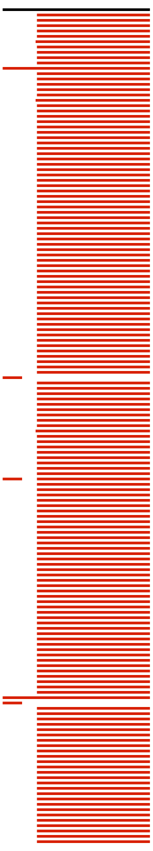

chr1:105634380-105641635

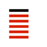

chr1:105814277-105826321

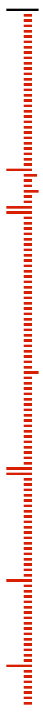

chr1:106110789-106116611

chr1:107023241-107025005

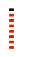

chr1:108535758-108539019

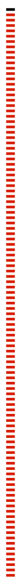

chr1:110025907-110058147

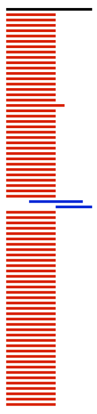

chr1:111179088-111189749

chr1:111629849-111637021

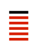

chr1:112489834-112507741

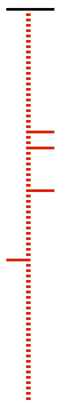

chr1:113551125-113553182

chr1:119900254-119916399

chr1:119916400-119953111

chr1:149603323-149664502

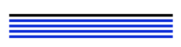

chr1:150670169-150702108

chr1:150821800-150857374

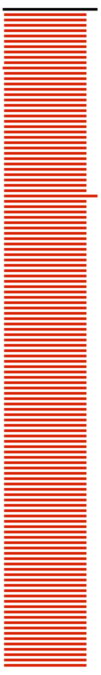

chr1:151028547-151035324

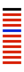

chr1:153455784-153467688

chr1:153927851-153929981

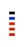

chr1:164451105-164460994

chr1:166355735-166358038

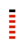

chr1:167500598-167524374

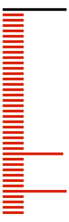

chr1:173062889-173068630

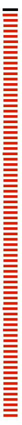

chr1:177220369-177245796

chr1:177597151-177599938

chr1:182046713-182052162

chr1:185564990-185567314

chr1:187353623-187359115

chr1:188236989-188253049

chr1:190095692-190133875

chr1:194994473-194997657

chr1:197378049-197380463

chr1:208148236-208150607

chr1:211071635-211077059

chr1:213560092-213565727

chr1:216484875-216489763

chr1:221083948-221090227

chr1:227883479-227886819

chr1:229598935-229609279

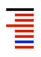

chr1:236918890-236922717

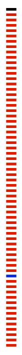

chr1:243505830-243513344

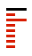

chr1:244095685-244103915

chr1:246672375-246727175

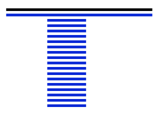

chr1:246798656-246880703

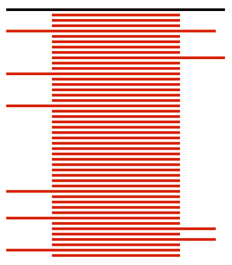

chr2:4191739-4201042

chr2:18037156-18056737

chr2:24454976-24469250

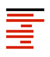

chr2:34552819-34590561

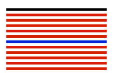

chr2:35831294-35841451

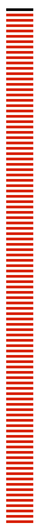

chr2:36185341-36193058

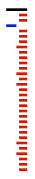

chr2:38727303-38730104

chr2:38809366-38825785

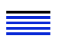

chr2:40780879-40803110

chr2:41091631-41099391

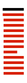

chr2:46549602-46551188

chr2:49389272-49399322

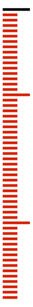

chr2:52595241-52635046

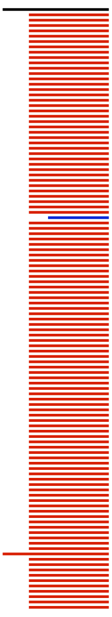

chr2:56149689-56241717

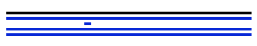

chr2:59801943-59806350

chr2:73729307-73787674

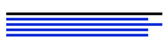

chr2:77830206-77850393

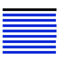

chr2:79184286-79195607

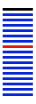

chr2:81665692-81669805

chr2:95094701-95099193

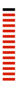

chr2:97475435-97535038

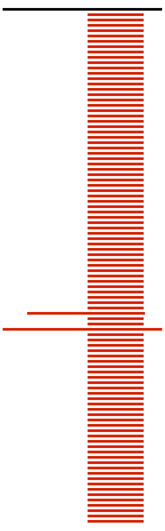

chr2:105714544-105717643

chr2:106239168-106253840

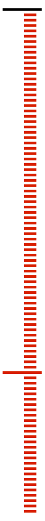

chr2:117489208-117508223

chr2:119762004-119766312

chr2:123192888-123200401

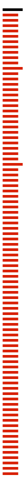

chr2:128538342-128543406

chr2:129354948-129356767

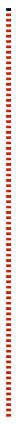

chr2:130510353-130542812

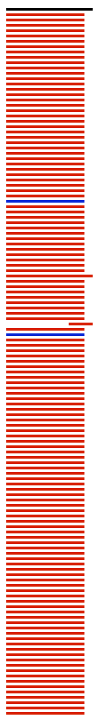

chr2:131809502-131812910

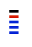

chr2:146578285-146583404

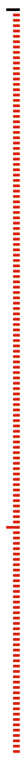

chr2:150739697-150745978

chr2:159418942-159438342

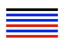

chr2:164351734-164353372

chr2:172543089-172548631

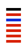

chr2:174301193-174305814

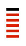

chr2:179775437-179787389

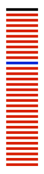

chr2:180126456-180130382

chr2:194295401-194306617

chr2:194426983-194446401

chr2:203608045-203610291

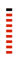

chr2:205366261-205391991

chr2:242643447-242738129

chr3:205022-207800

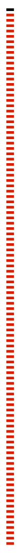

chr3:1658250-1666567

chr3:6626128-6630430

chr3:13682416-13684365

chr3:17504952-17519017

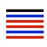

chr3:29013257-29016700

chr3:37957108-37969705

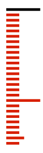

chr3:46776821-46824285

chr3:53003415-53016925

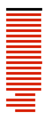

chr3:63108165-63110809

chr3:65164275-65185492

chr3:74230849-74234561

chr3:75655239-75678936

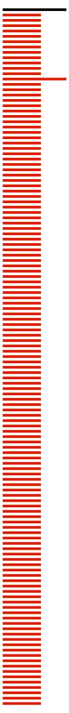

chr3:77910146-77924562

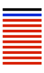

chr3:84781680-84794415

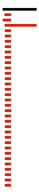

chr3:89477282-89503178

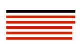

chr3:99370181-99402053

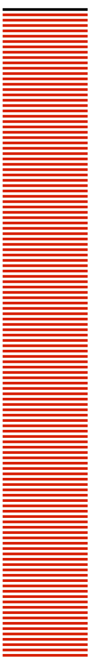

chr3:116143282-116151640

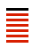

chr3:117689415-117698429

chr3:126926298-126941452

chr3:127156073-127158653

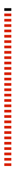

chr3:131240523-131294045

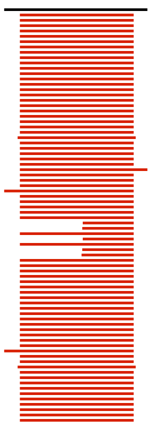

chr3:133475463-133478374

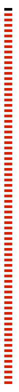

chr3:150444943-150450811

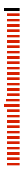

chr3:161839634-161844979

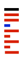

chr3:163699323-163709653

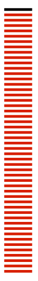

chr3:167573295-167576883

chr3:186584116-186592902

chr3:187888817-187900465

chr3:190842972-190847332

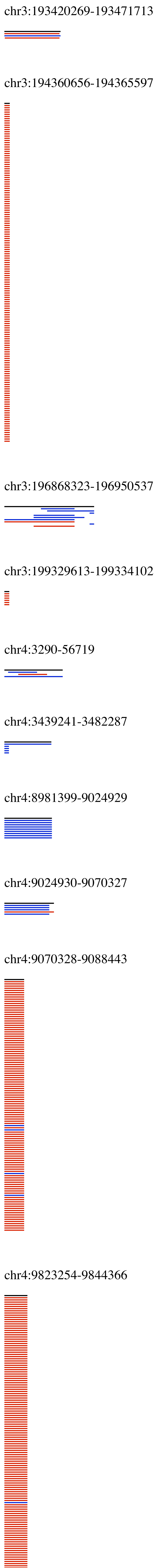
chr3:193420269-193471713
chr3:194360656-194365597
chr3:196868323-196950537
chr3:199329613-199334102
chr4:3290-56719
chr4:3439241-3482287
chr4:8981399-9024929
chr4:9024930-9070327
chr4:9070328-9088443
chr4:9823254-9844366

chr4:9881882-9884092

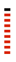

chr4:9999220-10009766

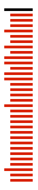

chr4:17153430-17177254

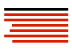

chr4:18697657-18733331

chr4:20977791-20985949

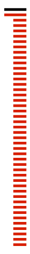

chr4:28895502-28907251

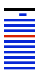

chr4:32122898-32126696

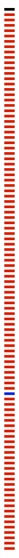

chr4:33055531-33062581

chr4:34455255-34528519

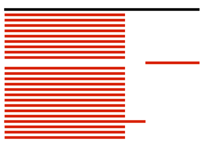

chr4:35524746-35527802

chr4:41152488-41161610

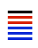

chr4:44018589-44025731

chr4:57942964-57953111

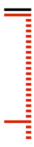

chr4:59655474-59673471

chr4:60007544-60013040

chr4:63352531-63354257

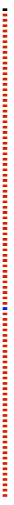

chr4:64370156-64400638

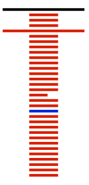

chr4:66485479-66488419

chr4:68964800-68970737

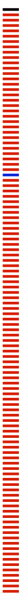

chr4:70158099-70162232

chr4:71262854-71285358

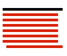

chr4:77508808-77511390

chr4:78001593-78006231

chr4:78495579-78500367

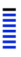

chr4:91506179-91510539

chr4:93631395-93637602

chr4:98390805-98404155

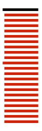

chr4:107162376-107165884

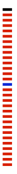

chr4:108285200-108294716

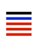

chr4:108731204-108733947

chr4:115393012-115406014

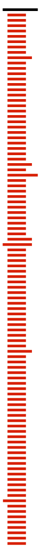

chr4:115727615-115729903

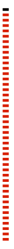

chr4:116392060-116395574

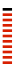

chr4:121743029-121762869

chr4:122501918-122504766

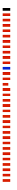

chr4:138308997-138314571

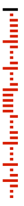

chr4:140452973-140457810

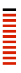

chr4:153008843-153010978

chr4:153010979-153014149

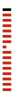

chr4:153209736-153212191

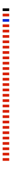

chr4:157187425-157194147

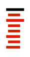

chr4:162075534-162151102

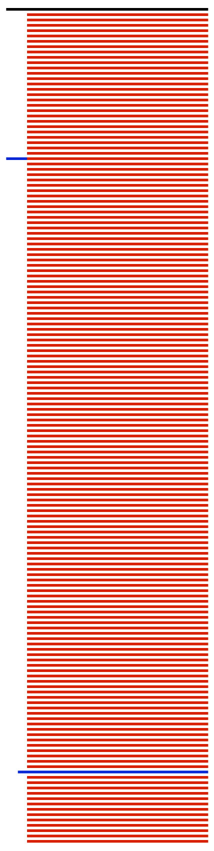

chr4:162413794-162424561

chr4:171504657-171509733

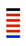

chr4:172611459-172614508

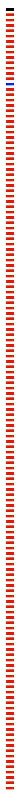

chr4:173661522-173665218

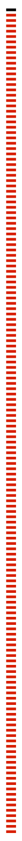

chr4:178705030-178723600

chr4:185223003-185226185

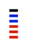

chr5:11181478-11184114

chr5:12843869-12868779

chr5:12868780-12879650

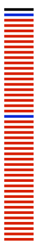

chr5:15767022-15773597

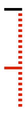

chr5:17563468-17644655

chr5:17644656-17730313

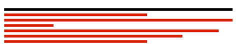

chr5:19315328-19323214

chr5:32142837-32215645

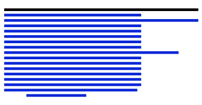

chr5:38180803-38184645

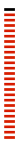

chr5:41267255-41280642

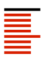

chr5:51465182-51467440

chr5:52440276-52445196

chr5:53703689-53729928

chr5:57361784-57369290

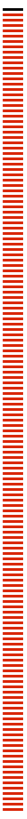

chr5:60280902-60312827

chr5:60608904-60617128

chr5:83985102-83990457

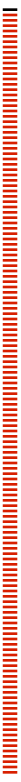

chr5:86151134-86154902

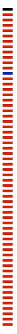

chr5:97142617-97146263

chr5:97512922-97517816

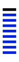

chr5:97961571-97963670

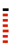

chr5:98117485-98123869

chr5:106257572-106261016

chr5:110937230-110938944

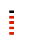

chr5:113979007-114001657

chr5:114734342-114748984

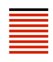

chr5:114784536-114787066

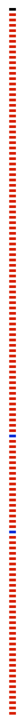

chr5:135329914-135362750

chr5:140204020-140223940

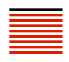

chr5:143386876-143390529

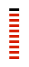

chr5:150185693-150198797

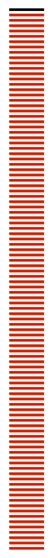

chr5:151495579-151498544

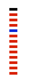

chr5:152668371-152673136

chr5:155409350-155415307

chr5:160471835-160482276

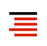

chr5:163153787-163155953

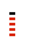

chr5:174368627-174371849

chr5:177090005-177165211

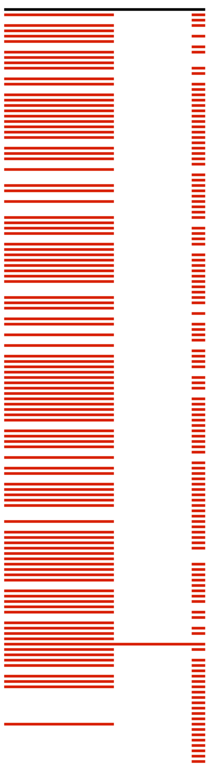

chr5:178042581-178045769

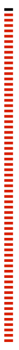

chr5:180304774-180365870

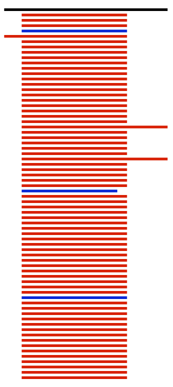

chr6:11182288-11185184

chr6:26812795-26907945

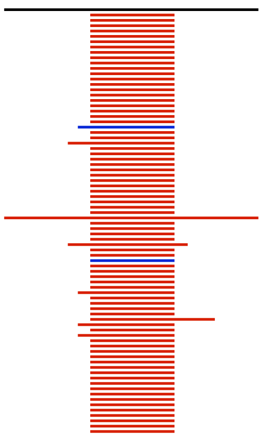

chr6:29192066-29269414

chr6:29945171-30029106

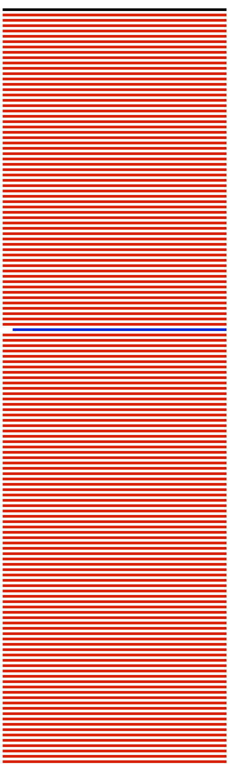

chr6:31385665-31404430

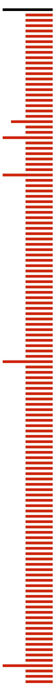

chr6:31445851-31448789

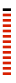

chr6:31467630-31559455

chr6:32700999-32710085

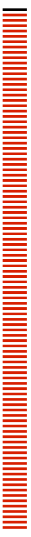

chr6:34046721-34050683

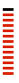

chr6:44621319-44622929

chr6:49039688-49043159

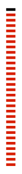

chr6:65768120-65770513

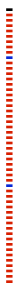

chr6:66427073-66463931

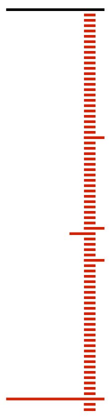

chr6:67064983-67101187

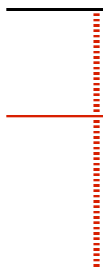

chr6:67830888-67861414

chr6:74648953-74658138

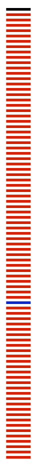

chr6:74766429-74773741

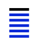

chr6:77070276-77084501

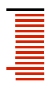

chr6:77496587-77509523

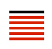

chr6:79025784-79091904

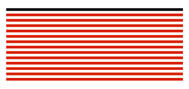

chr6:79577781-79585293

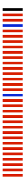

chr6:81332484-81351822

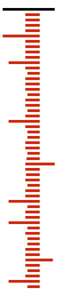

chr6:93632587-93635351

chr6:95597240-95650995

chr6:103840867-103868754

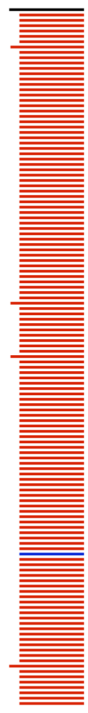

chr6:121014994-121044115

chr6:126225385-126228469

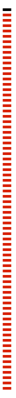

chr6:134592448-134598896

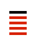

chr6:140421912-140440964

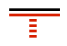

chr6:154163673-154169933

chr6:165647807-165651918

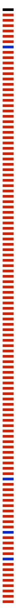

chr6:169249708-169260864

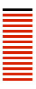

chr7:3568378-3597190

chr7:4299801-4355147

chr7:6820635-6830608

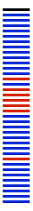

chr7:16315288-16369548

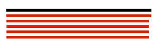

chr7:19144600-19153324

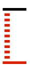

chr7:23107172-23110722

chr7:26108917-26111938

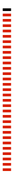

chr7:56729607-56736308

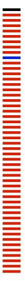

chr7:61477137-61488622

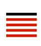

chr7:61933205-62007269

chr7:64697484-64744239

chr7:66266764-66282667

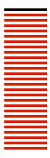

chr7:70058925-70064077

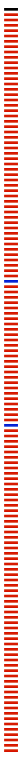

chr7:82553896-82555900

chr7:86072120-86082990

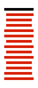

chr7:90023270-90058767

chr7:90864344-90885502

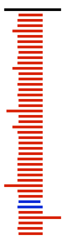

chr7:97090174-97096105

chr7:100167180-100170778

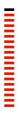

chr7:102139082-102145851

chr7:104250981-104263771

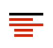

chr7:109220983-109241507

chr7:112301356-112305297

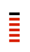

chr7:115370689-115389179

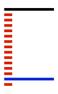

chr7:118479927-118481573

chr7:120847107-120869296

chr7:125832159-125834117

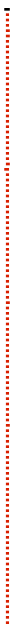

chr7:133428969-133459332

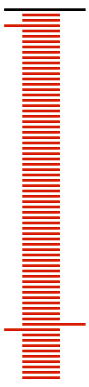

chr7:139650508-139654937

chr7:141416112-141438377

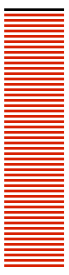

chr7:141693581-141715788

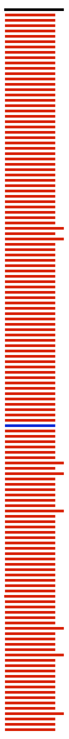

chr7:141891589-141909210

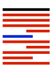

chr7:142155609-142179754

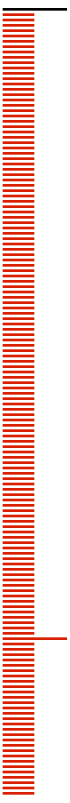

chr7:143341029-143342084

chr7:149425988-149435586

chr7:149916734-149932502

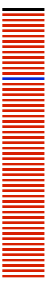

chr7:152229180-152247032

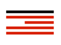

chr7:154024104-154031766

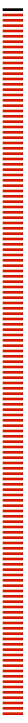

chr7:156084551-156086940

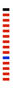

chr7:157031127-157043601

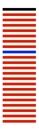

chr7:157810312-157824926

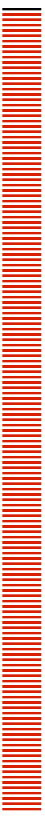

chr8:1346118-1348244

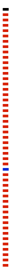

chr8:2067801-2069206

chr8:3944372-3949289

chr8:4048334-4058846

chr8:5586134-5594648

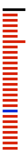

chr8:5841704-5938256

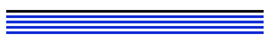

chr8:6095442-6101681

chr8:6109511-6111533

chr8:12025135-12072406

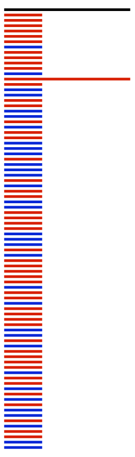

chr8:12593116-12606321

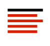

chr8:12693099-12696897

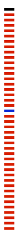

chr8:13854600-13859608

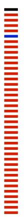

chr8:15445295-15457818

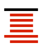

chr8:15738520-15780728

chr8:24201375-24207011

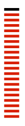

chr8:25030360-25040250

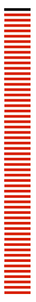

chr8:32802640-32807955

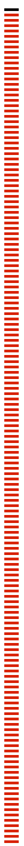

chr8:47646713-47655711

chr8:50622571-50695376

chr8:51194564-51195976

chr8:55371599-55373373

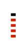

chr8:58279104-58292579

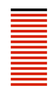

chr8:58520879-58529512

chr8:68710013-68712760

chr8:72374735-72379153

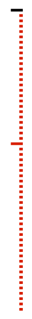

chr8:85425438-85431221

chr8:100137111-100170208

chr8:102057367-102061693

chr8:102689782-102694888

chr8:107927695-107931446

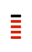

chr8:112354518-112370428

chr8:115700470-115723944

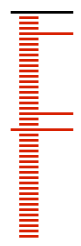

chr8:117699864-117702442

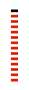

chr8:120223965-120228627

chr8:127346504-127348631

chr8:130185878-130197300

chr8:130211860-130213708

chr8:133060267-133063758

chr8:135130437-135135890

chr8:136692574-136694147

chr8:142923949-142931358

chr8:144776300-144778951

chr9:4515758-4527834

chr9:5301567-5327707

chr9:8605659-8610138

chr9:11957033-11965492

chr9:16061336-16064727

chr9:20789776-20800158

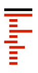

chr9:23353115-23363484

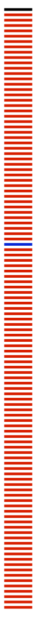

chr9:28037588-28040691

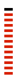

chr9:29084549-29087680

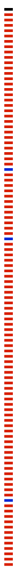

chr9:37475300-37505711

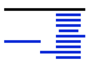

chr9:41962506-41975187

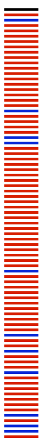

chr9:43177963-43255665

chr9:66405631-66439058

chr9:67645568-67678367

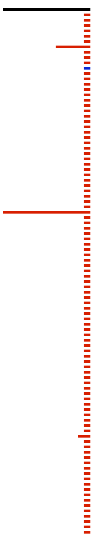

chr9:71287475-71310097

chr9:78746701-78749156

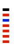

chr9:85704275-85707363

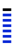

chr9:93442089-93443266

chr9:98700200-98729161

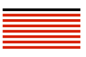

chr9:112064610-112067990

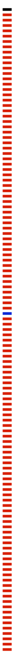

chr9:114894172-114931716

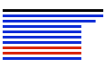

chr9:128015132-128016523

chr9:133250747-133255525

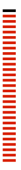

chr9:134926833-134933034

chr9:134933035-134947261

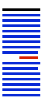

chr9:137353926-137356233

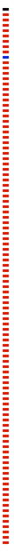

chr9:140145139-140152969

chr9:140184460-140211215

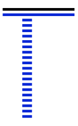

chr10:123551-133255

chr10:20890118-20893540

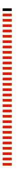

chr10:24201315-24206056

chr10:24415269-24419045

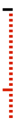

chr10:26726161-26729773

chr10:27265910-27268479

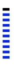

chr10:45392878-45396241

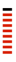

chr10:56122955-56138443

chr10:56353096-56362605

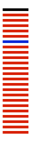

chr10:56849158-56853055

chr10:58186381-58196843

chr10:58516320-58531511

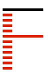

chr10:58572176-58606915

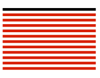

chr10:61034136-61035270

chr10:66975075-66999412

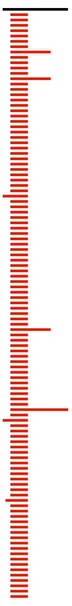

chr10:77927067-77930591

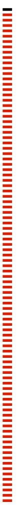

chr10:79086085-79112590

chr10:81053880-81055405

chr10:83874640-83878647

chr10:84033480-84048907

chr10:84400707-84421820

chr10:84702878-84707152

chr10:87791026-87798355

chr10:90932947-90938070

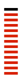

chr10:91988396-91992471

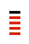

chr10:107047028-107049160

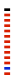

chr10:114102065-114106829

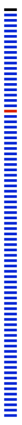

chr10:124331524-124347716

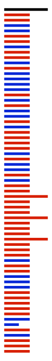

chr11:1837832-1917776

chr11:4923637-4934958

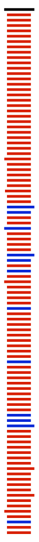

chr11:5228247-5230232

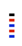

chr11:5478747-5480169

chr11:5739501-5768936

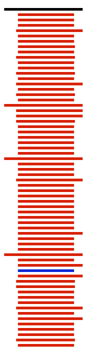

chr11:5828813-5892086

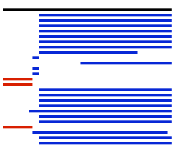

chr11:7769616-7798159

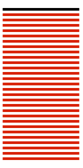

chr11:9391796-9397985

chr11:18905648-18918939

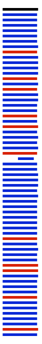

chr11:24400825-24406833

chr11:30129311-30137019

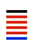

chr11:42769727-42774615

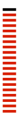

chr11:42926159-42927744

chr11:48843099-48849795

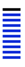

chr11:49667437-49714078

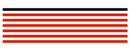

chr11:49716131-49717264

chr11:51209566-51213821

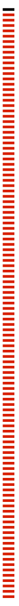

chr11:54458221-54530469

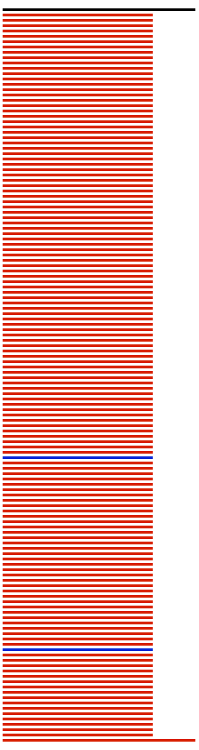

chr11:55130608-55209585

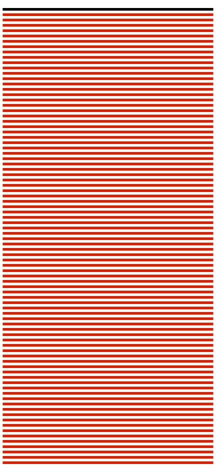

chr11:57608407-57612191

chr11:58556950-58618670

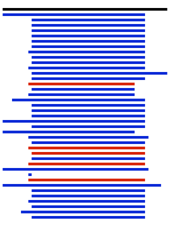

chr11:80575850-80583269

chr11:81176206-81189918

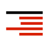

chr11:81189919-81194913

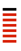

chr11:90207835-90210585

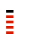

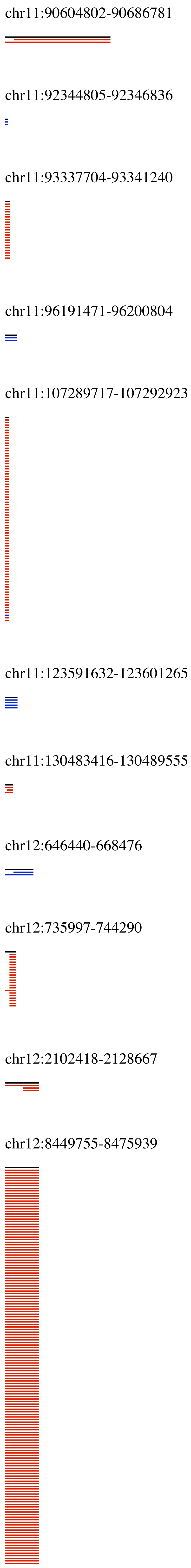
chr11:90604802-90686781
chr11:92344805-92346836
chr11:93337704-93341240
chr11:96191471-96200804
chr11:107289717-107292923
chr11:123591632-123601265
chr11:130483416-130489555
chr12:646440-668476
chr12:735997-744290
chr12:2102418-2128667
chr12:8449755-8475939

chr12:10474634-10487386

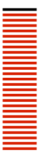

chr12:11106639-11143141

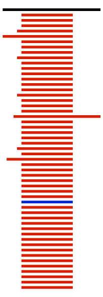

chr12:11372533-11445145

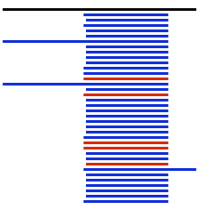

chr12:12428764-12433138

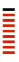

chr12:27986760-27988911

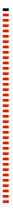

chr12:28656614-28658887

chr12:30128764-30135252

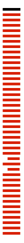

chr12:33191058-33199856

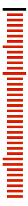

chr12:33270866-33286685

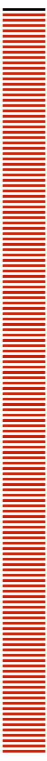

chr12:34131258-34155356

chr12:36947182-36955164

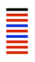

chr12:39103864-39107745

chr12:44192107-44195804

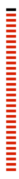

chr12:60488837-60497087

chr12:69155719-69167195

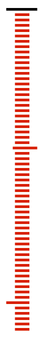

chr12:69785308-69796522

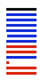

chr12:79349306-79352244

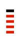

chr12:82687421-82689372

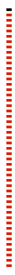

chr12:85225213-85236932

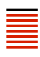

chr12:86055044-86057392

chr12:91161059-91171445

chr12:98518469-98532452

chr12:116533707-116535910

chr12:126141646-126151541

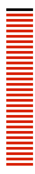

chr12:127796462-127798498

chr12:130360121-130391708

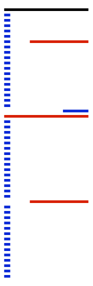

chr12:130680837-130686668

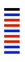

chr13:21994827-22004855

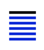

chr13:22443593-22454441

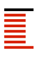

chr13:24407846-24413343

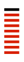

chr13:25108823-25124911

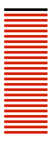

chr13:31429924-31436423

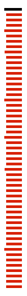

chr13:32346889-32352685

chr13:36970036-36986387

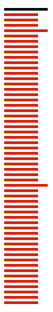

chr13:42313804-42322187

chr13:42497548-42501093

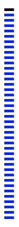

chr13:51969153-52043659

chr13:56842712-56848581

chr13:57488282-57495031

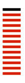

chr13:63122789-63134693

chr13:63227094-63303323

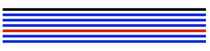

chr13:68149981-68166243

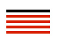

chr13:71375556-71378557

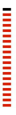

chr13:78100453-78107836

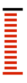

chr13:88594853-88637416

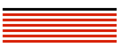

chr13:103073621-103077481

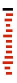

chr13:109470103-109476776

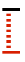

chr14:19609203-19665855

chr14:20034116-20041624

chr14:20419446-20483195

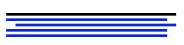

chr14:21119983-21125597

chr14:22164681-22169049

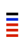

chr14:23499138-23570841

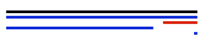

chr14:25832761-25849239

chr14:35741777-35744784

chr14:39940729-39942282

chr14:40541848-40567355

chr14:40680246-40727099

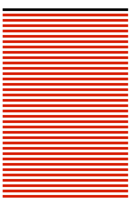

chr14:42665709-42669587

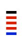

chr14:43544413-43602046

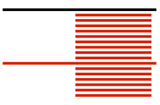

chr14:64804244-64827501

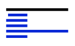

chr14:69088121-69092220

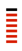

chr14:73070876-73090732

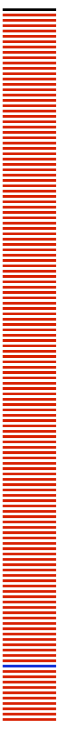

chr14:73101243-73120017

chr14:81705838-81708582

chr14:105952397-105989694

chr15:18390155-18476969

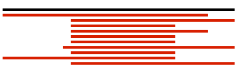

chr15:20658550-20676330

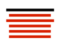

chr15:22029857-22065565

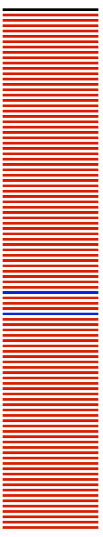

chr15:22329509-22343467

chr15:30291556-30299512

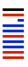

chr15:31693429-31696262

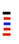

chr15:37375518-37379480

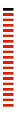

chr15:45992538-46001995

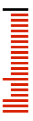

chr15:54577995-54588269

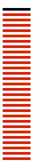

chr15:74127925-74131944

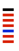

chr15:74678296-74682830

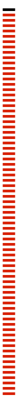

chr15:78310026-78313665

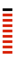

chr15:82331742-82334554

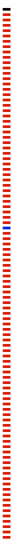

chr15:84312709-84322841

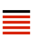

chr16:7130898-7174339

chr16:20453641-20464612

chr16:34872574-34916829

chr16:54352452-54379945

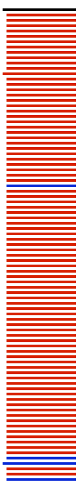

chr16:54387058-54425940

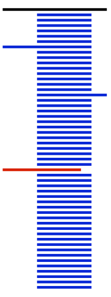

chr16:58640103-58654487

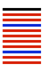

chr16:72953795-73014102

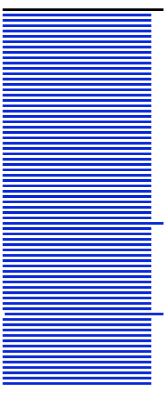

chr16:74096909-74115583

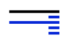

chr16:74115584-74133500

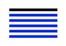

chr16:74565193-74578672

chr16:75224186-75226991

chr16:75758984-75780371

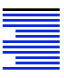

chr16:78451145-78488148

chr16:82447180-82449270

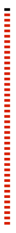

chr16:86671164-86672892

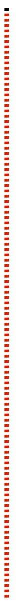

chr17:14984651-14995227

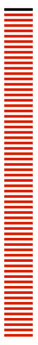

chr17:15483886-15487515

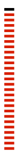

chr17:15591355-15614730

chr17:16598056-16665025

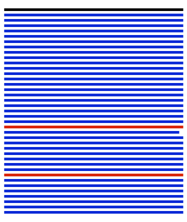

chr17:18229719-18296116

chr17:18539508-18543257

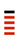

chr17:36641086-36647295

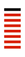

chr17:36666936-36686792

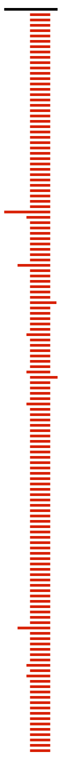

chr17:36786395-36790200

chr17:36920703-36936394

chr17:41006741-41022689

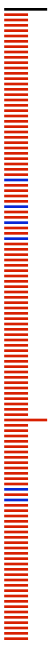

chr17:72728774-72743351

chr18:38133-68539

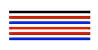

chr18:283519-294262

chr18:36514418-36519387

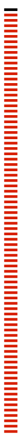

chr18:49461207-49464638

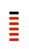

chr18:56068921-56073194

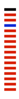

chr18:63104629-63119574

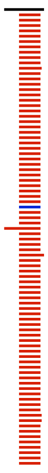

chr18:64893256-64909246

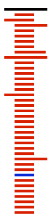

chr18:65358207-65368807

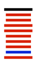

chr18:74763700-74765854

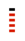

chr19:5461239-5464062

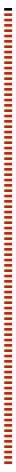

chr19:8256197-8271067

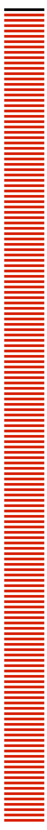

chr19:9135672-9139121

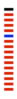

chr19:24252658-24256797

chr19:34029192-34031422

chr19:40537990-40553688

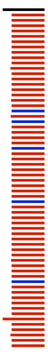

chr19:46030675-46072786

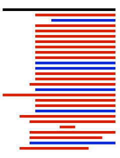

chr19:46143504-46205185

chr19:50542545-50583764

chr19:55949062-55957853

chr19:56823448-56840009

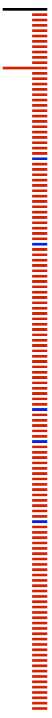

chr19:58008152-58055950

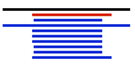

chr19:58210559-58244245

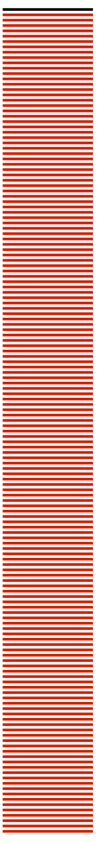

chr19:58570456-58645684

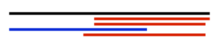

chr19:59422012-59433131

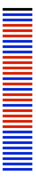

chr19:59473655-59498495

chr19:59843208-59868103

chr19:59989695-60065630

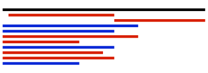

chr19:60959856-60980753

chr19:61896070-61903323

chr20:1505190-1546336

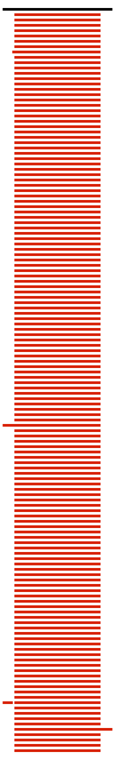

chr20:40656476-40726214

chr20:45210204-45224368

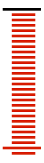

chr20:59002420-59023596

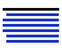

chr20:62425139-62426597

chr21:13368583-13374732

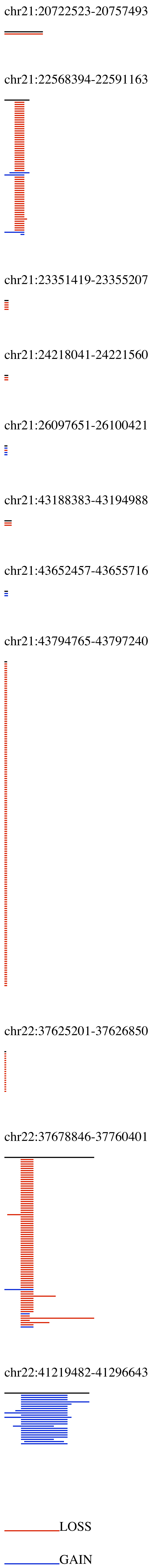
chr21:20722523-20757493
chr21:22568394-22591163
chr21:23351419-23355207
chr21:24218041-24221560
chr21:26097651-26100421
chr21:43188383-43194988
chr21:43652457-43655716
chr21:43794765-43797240
chr22:37625201-37626850
chr22:37678846-37760401
chr22:41219482-41296643
LOSS
GAIN
