# Supplementary material for: A Map of Copy Number Variations in Chinese Populations
Source: PLoS One. 2011 Nov 7;6(11):e27341. doi: 10.1371/journal.pone.0027341 (PMC3210162; doi:10.1371/journal.pone.0027341)
Supplement: Figure S7 — The state of Individual CNVs on non-singleton CNVRs (CNVR length> = 100 kb). Black bars denote CNVR, and below which each bar denote a CNV call in one individual. Red and blue represent deletion and duplication, respectively. (PDF) [file pone.0027341.s007.pdf]

chr1:554484-840550

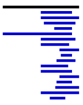

chr1:1617778-1750923

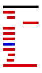

chr1:12743662-13379464

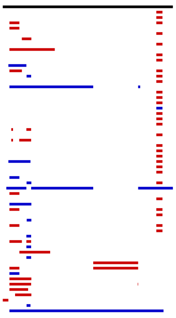

chr1:16741950-17150527

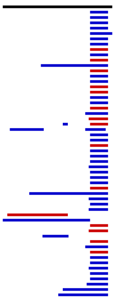

chr1:17857036-18485275

chr1:25434674-25535931

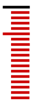

chr1:60888686-61041374

chr1:65630676-65999483

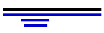

chr1:103526436-103819266

chr1:103910761-104128012

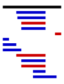

chr1:105835455-106057949

chr1:119490013-119780209

chr1:142693888-143049286

chr1:144337063-144724627

chr1:146780414-147788470

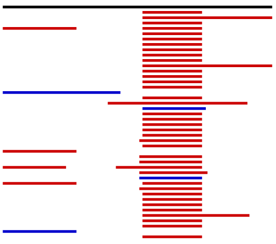

chr1:159763524-159906183

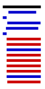

chr1:187106397-187350656

chr1:187592364-187814261

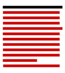

chr1:194997658-195183422

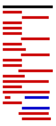

chr1:241334731-241457448

chr2:7135336-7340939

chr2:13455187-13908456

chr2:13964040-14252587

chr2:87104007-87805782

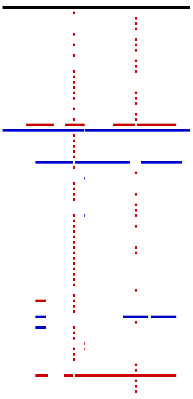

chr2:89395525-89912071

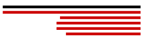

chr2:97094860-97391786

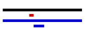

chr2:110147004-110603665

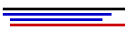

chr2:116257824-116391820

chr2:130660292-130874329

chr2:216861288-217162631

chr2:232901812-233018302

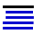

chr3:20310916-20882646

chr3:20949623-21095913

chr3:28307024-28438601

chr3:49346010-49489741

chr3:50317228-50523899

chr3:68614345-68990246

chr3:125782638-126494752

chr3:163995351-164109297

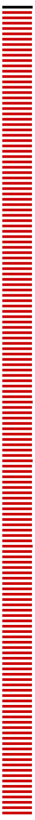

chr3:196964365-197137370

chr4:58821-212068

chr4:22293290-22405586

chr4:29159382-29309515

chr4:45619591-45891135

chr4:56879617-57069085

chr4:57194023-57392352

chr4:64918291-65128774

chr4:69043083-69204004

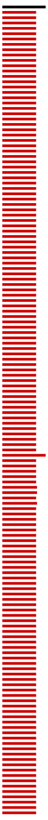

chr4:70162233-70298973

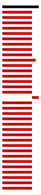

chr4:87910022-88097650

chr4:99279567-99383896

chr4:145140024-145252346

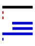

chr4:152053479-152252680

chr4:178204460-178379530

chr4:190384723-190701251

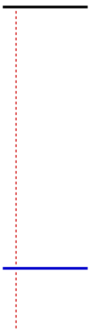

chr4:191146321-191261904

chr5:751371-886627

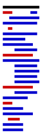

chr5:13226664-13421282

chr5:45789691-45916139

chr5:46227005-46349398

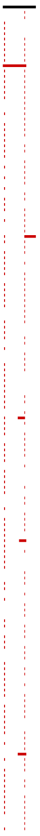

chr5:107598684-107834240

chr5:107878643-108109865

chr5:149562665-149684402

chr5:164273821-164424234

chr5:178662194-178863916

chr6:198272-332659

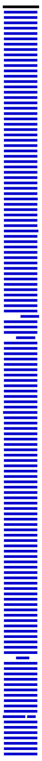

chr6:26220383-26413386

chr6:32539530-32681749

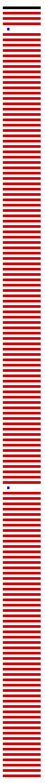

chr6:58248259-58882675

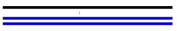

chr6:81705925-81878449

chr6:95330385-95590059

chr6:162449123-162953768

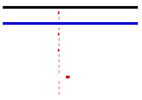

chr7:71798-326228

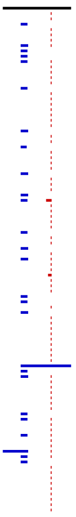

chr7:5293964-5469490

chr7:19379124-19511836

chr7:19619636-19978999

chr7:53433670-53558241

chr7:62315457-62467318

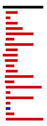

chr7:63396930-63500236

chr7:63508459-63622160

chr7:64231500-64605202

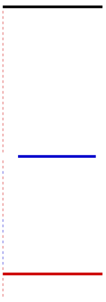

chr7:73141429-73355380

chr7:74998062-75192118

chr7:75202175-75373301

chr7:76163693-76439521

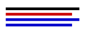

chr7:96039672-96178245

chr7:110719406-110900827

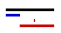

chr7:112096428-112232697

chr7:113037047-113216830

chr7:113220076-113528731

chr7:142929090-143203219

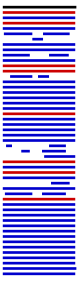

chr7:143527185-143697880

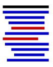

chr7:149190618-149425987

chr7:151477446-151580599

chr7:153130910-153302383

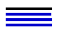

chr8:2113807-2382517

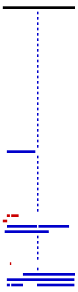

chr8:3674807-3777514

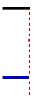

chr8:6891605-6998485

chr8:7157884-8055448

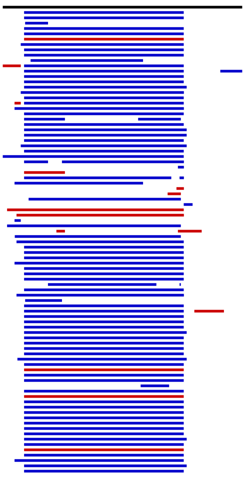

chr8:12225652-12536010

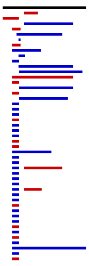

chr8:18846542-18953095

chr8:19035043-19160673

chr8:35813728-36497057

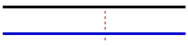

chr8:39349340-39508281

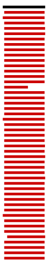

chr8:53428165-53715799

chr8:83489145-83705565

chr8:112064480-112212410

chr8:121483185-121867498

chr8:142582575-142707176

chr8:146165959-146268959

chr9:11998577-12138728

chr9:13215407-13508433

chr9:30327953-30514025

chr9:38994848-39191451

chr9:39232277-39800645

chr9:40466154-40901224

chr9:43255666-44795733

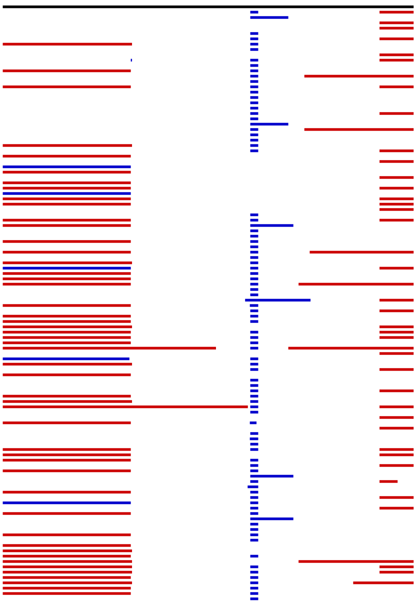

chr9:45270026-45953932

chr9:68055004-68890044

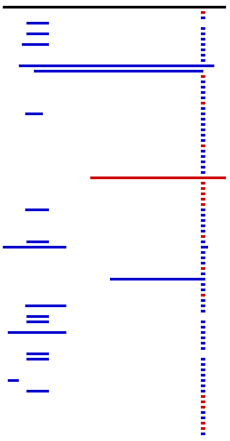

chr9:68905449-69114783

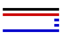

chr9:69114784-69267842

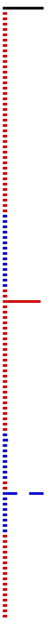

chr9:79934142-80339907

chr9:118291339-118421106

chr10:180384-292648

chr10:861038-1091044

chr10:41753546-42259374

chr10:46111165-47241036

chr10:59242014-59441474

chr10:62076611-62177209

chr10:66363458-66503261

chr10:67650978-67756487

chr10:67838185-68185158

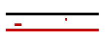

chr10:81475459-81593474

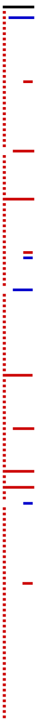

chr10:88987279-89098396

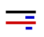

chr10:135092863-135227268

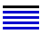

chr10:135250508-135356694

chr11:4206589-4315864

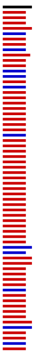

chr11:50952568-51185373

chr11:51231886-51421003

chr11:54530469-54796378

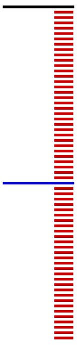

chr11:66879237-66993741

chr11:67239223-67488890

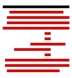

chr11:96783683-97433009

chr11:106558245-106732690

chr11:129034292-129168247

chr12:7895692-8028989

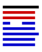

chr12:9496442-9626237

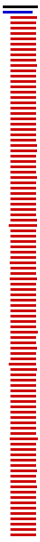

chr12:20652661-20785273

chr12:20793555-21078914

chr12:62219230-62422921

chr12:65622693-65806729

chr12:86502634-86639183

chr12:122406526-122552874

chr13:18041824-18165293

chr13:56610801-56786968

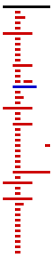

chr13:64890871-65055390

chr14:18072124-18195701

chr14:18452209-19494766

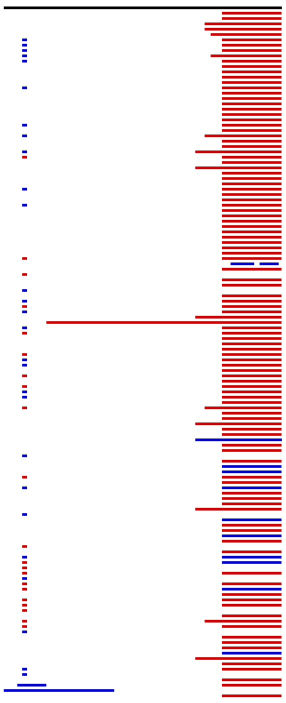

chr14:47449986-47725196

chr14:105100682-105295569

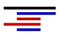

chr14:105609107-105854089

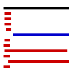

chr15:18506386-20232439

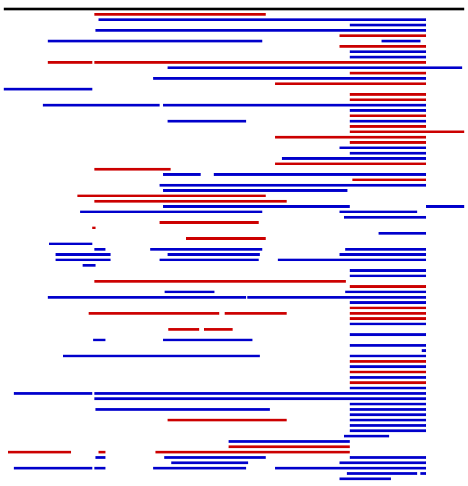

chr15:22071426-22329508

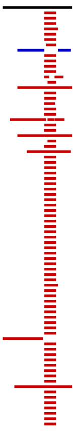

chr15:28173703-28609050

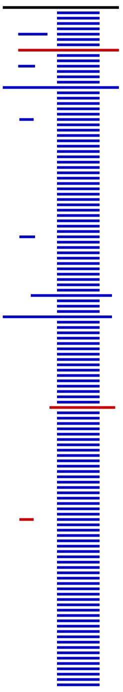

chr15:28688170-28875769

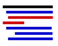

chr15:29798781-30231488

chr15:30318608-30647150

chr15:32459378-32650015

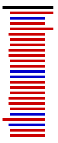

chr15:41672410-41783180

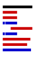

chr15:88433614-88579397

chr16:470380-593863

chr16:2530025-2681813

chr16:14796096-15023758

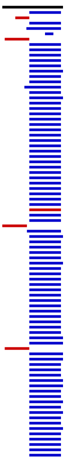

chr16:16260667-16706164

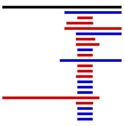

chr16:18336886-18675619

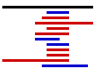

chr16:21270906-21513685

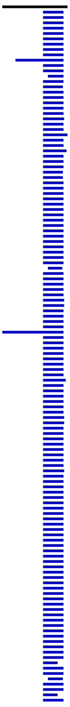

chr16:21526249-21650633

chr16:22349475-22465432

chr16:22465433-22620480

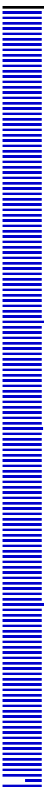

chr16:30134444-30419276

chr16:31873117-33208394

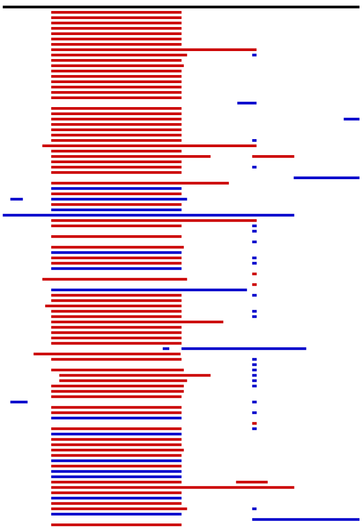

chr16:33208395-33683586

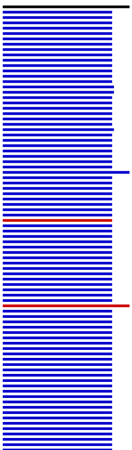

chr16:34324072-34614568

chr16:68607297-68770545

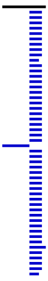

chr16:76011878-76174547

chr16:76188266-76412349

chr16:76435072-76637919

chr16:76645062-77052862

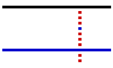

chr17:4157041-4326327

chr17:18296117-18415371

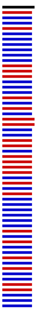

chr17:18876248-19070824

chr17:21272823-21470495

chr17:21473462-21859450

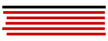

chr17:31459625-31610407

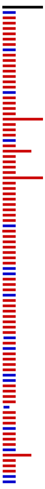

chr17:41521619-41719991

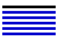

chr17:41750187-42143377

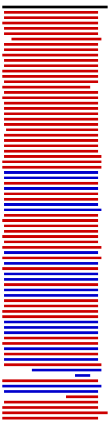

chr17:68480897-68633353

chr18:14157402-14342869

chr18:14574147-14731650

chr19:918584-1028701

chr19:2356116-2510841

chr19:10161930-10346581

chr19:14374781-14523408

chr19:20386147-20513302

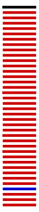

chr19:22128734-22999226

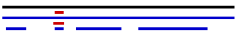

chr19:47978604-48482591

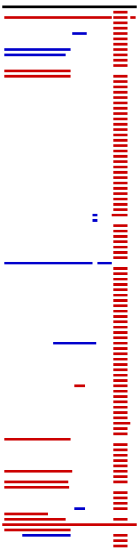

chr19:52437315-52551397

chr20:28034013-28151003

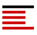

chr21:9758742-10197783

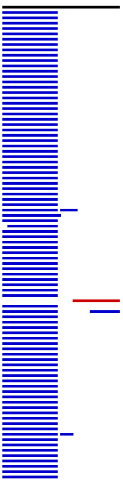

chr21:13374733-13483809

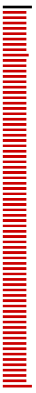

chr21:16472786-16662257

chr22:14482342-15037301

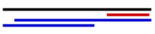

chr22:17173947-17386125

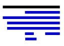

chr22:20055998-20175294

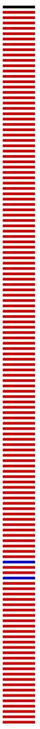

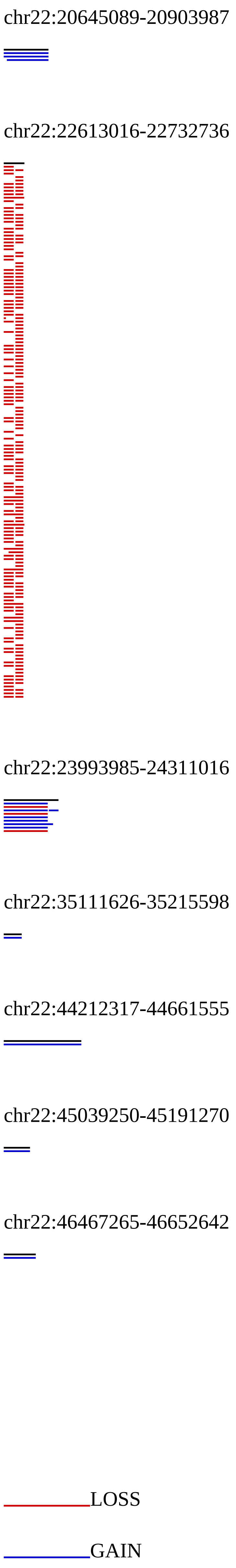
chr22:20645089-20903987
chr22:22613016-22732736
chr22:23993985-24311016
chr22:35111626-35215598
chr22:44212317-44661555
chr22:45039250-45191270
chr22:46467265-46652642
LOSS
GAIN
