# Supplementary material for: A Map of Copy Number Variations in Chinese Populations
Source: PLoS One. 2011 Nov 7;6(11):e27341. doi: 10.1371/journal.pone.0027341 (PMC3210162; doi:10.1371/journal.pone.0027341)
Supplement: Figure S8 — CNV sharing. Venn diagram of (A).Deletion, (C).Duplication and (E).Multi-allelic CNVs sharing results among African, Asian and European groups (each group with sample-size 425). Venn diagram of (B).Deletion, (D).Duplication and (F).Multi-allelic CNVs sharing results among Han Chinese, Chinese minority and Japanese groups (each group with sample-size 75). (PDF) [file pone.0027341.s008.pdf]

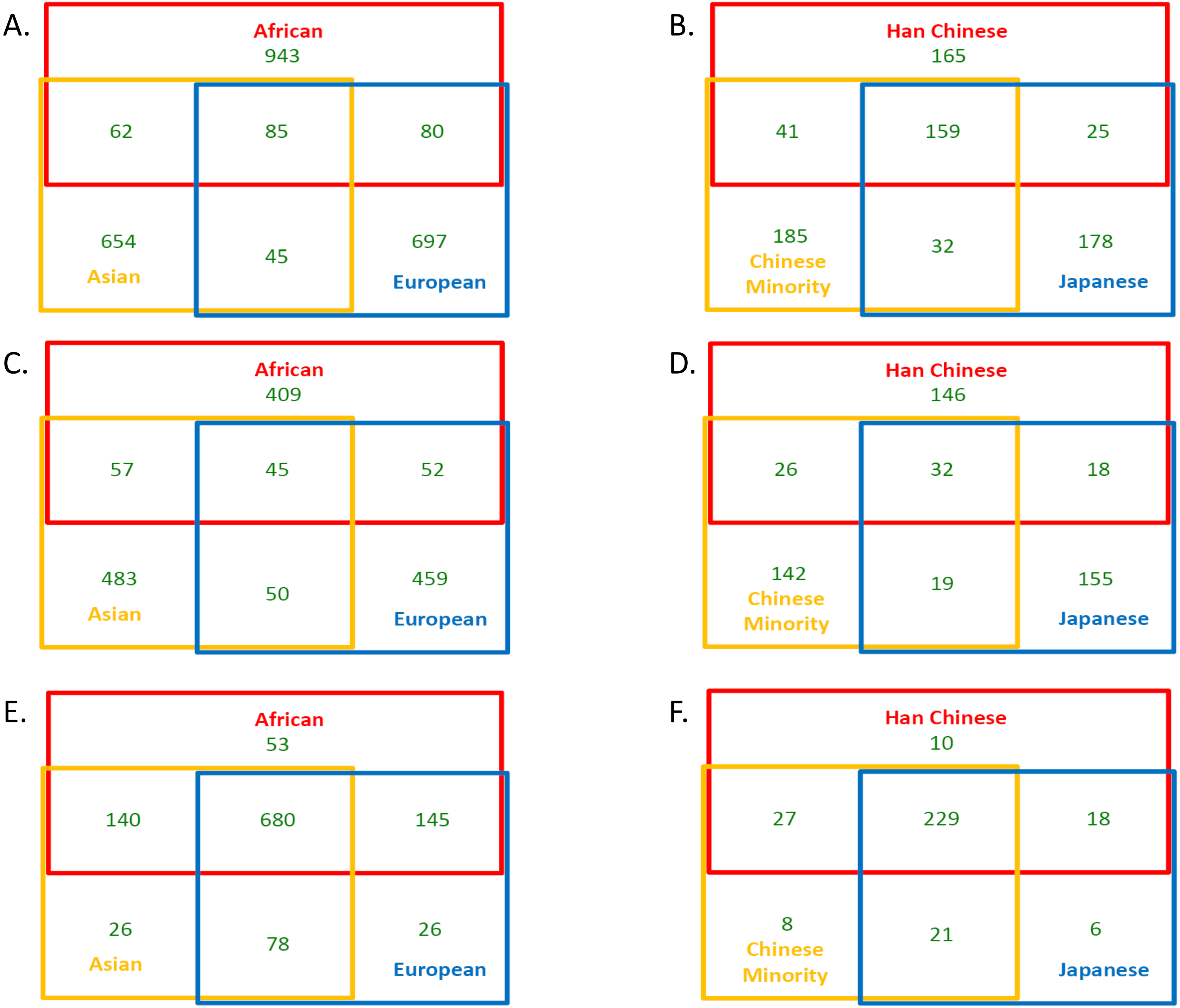
A.
African
943
62
85
80
654
Asian
45
697
European
B.
Han Chinese
165
41
159
25
185
Chinese Minority
32
178
Japanese
C.
African
409
57
45
52
483
Asian
50
459
European
D.
Han Chinese
146
26
32
18
142
Chinese Minority
19
155
Japanese
E.
African
53
140
680
145
26
Asian
78
26
European
F.
Han Chinese
10
27
229
18
8
Chinese Minority
21
6
Japanese
